# Neural-Induced Human Adipose Tissue-Derived Stem Cells Conditioned Medium Ameliorates Rotenone-Induced Toxicity in SH-SY5Y Cells

**DOI:** 10.3390/ijms22052322

**Published:** 2021-02-26

**Authors:** Mahesh Ramalingam, Sujeong Jang, Han-Seong Jeong

**Affiliations:** Department of Physiology, Chonnam National University Medical School, Hwasun 58128, Jeollanam-do, Korea

**Keywords:** Parkinson’s disease, α-synuclein, rotenone, regeneration, mesenchymal stem cells

## Abstract

Parkinson’s disease (PD) is an age-related neurodegenerative disease (NDD) characterized by the degenerative loss of dopaminergic neurons in the substantia nigra along with aggregation of α-synuclein (α-syn). Neurogenic differentiation of human adipose-derived stem cells (NI-hADSCs) by supplementary factors for 14 days activates different biological signaling pathways. In this study, we evaluated the therapeutic role of NI-hADSC-conditioned medium (NI-hADSC-CM) in rotenone (ROT)-induced toxicity in SH-SY5Y cells. Increasing concentrations of ROT led to decreased cell survival at 24 and 48 h in a dose- and time-dependent manner. Treatment of NI-hADSC-CM (50% dilution in DMEM) against ROT (0.5 μM) significantly increased the cell survival. ROT toxicity decreased the expression of tyrosine hydroxylase (TH). Western blot analysis of the Triton X-100-soluble fraction revealed that ROT significantly decreased the oligomeric, dimeric, and monomeric phosphorylated Serine129 (p-S129) α-syn, as well as the total monomeric α-syn expression levels. ROT toxicity increased the oligomeric, but decreased the dimeric and monomeric p-S129 α-syn expression levels. Total α-syn expression (in all forms) was increased in the Triton X-100-insoluble fraction, compared to the control. NI-hADSC-CM treatment enhanced the TH expression, stabilized α-syn monomers, reduced the levels of toxic insoluble p-S129 α-syn, improved the expression of neuronal functional proteins, regulated the Bax/Bcl-2 ratio, and upregulated the expression of pro-caspases, along with PARP-1 inactivation. Moreover, hADSC-CM treatment decreased the cell numbers and have no effect against ROT toxicity on SH-SY5Y cells. The therapeutic effects of NI-hADSC-CM was higher than the beneficial effects of hADSC-CM on cellular signaling. From these results, we conclude that NI-hADSC-CM exerts neuroregenerative effects on ROT-induced PD-like impairments in SH-SY5Y cells.

## 1. Introduction

Neurodegenerative diseases (NDDs) including Alzheimer’s disease (AD), Parkinson’s disease (PD), Huntington’s disease (HD), and amyotrophic lateral sclerosis (ALS), are characterized by the chronic loss of different neuronal subtypes [1]. PD is an age-related NDD characterized by the progressive loss of dopaminergic (DAergic) neurons in the substantia nigra (SN) pars compacta (SNpc) and the formation of misfolded protein aggregates [2]. Reduction of the rate-limiting enzyme in the biosynthesis of catecholamine neurotransmitters, tyrosine hydroxylase (TH), results in the loss of dopamine in the basal ganglia, an area of the brain responsible for fine motor control [3]. Progressive and abnormal protein aggregation results in the accumulation of intracellular α-synuclein (α-syn) inclusions known as Lewy bodies (LBs) and Lewy neurites (LNs) composed of aggregated α-syn fibrils, which seems to be associated with PD [2].

Rotenone (ROT), a piscicide extracted from the roots and plants belonging to the genera *Lonchocarpus* and *Derris*, is highly lipophilic and can easily cross all biological membranes, including the blood-brain barrier (BBB). ROT inhibits the activity of the mitochondrial electron transport chain (ETC) complex 1 (EC 7.1.1.2; reduced nicotinamide adenine dinucleotide ubiquinone reductase, NADH ubiquinone oxidoreductase, and Type I NADH dehydrogenase), leading to reduced ATP production and formation of reactive oxygen species (ROS), which can induce oxidative stress and impair oxidative phosphorylation [4]. Indeed, ROT has been reported for its ability to induce the formation of α-syn-positive cytoplasmic inclusions resembling LBs in nigral neurons, accompanied by PD-like neurodegeneration [5].

α-syn is a relatively small ubiquitous protein consisting of 140 amino acids; it is highly expressed in the presynaptic structures of the brain. α-syn exists as species with multiple molecular weights, including monomeric, dimeric, oligomeric, and fibrillary structures [6]. Monomeric α-syn has an amphipathic N-terminus, a hydrophobic central region (non-amyloid component), and an acidic C-terminus. In native conditions, monomeric α-syn is intrinsically soluble and capable of binding to membrane phospholipids; however, the aggregation process of α-syn involves the formation of several intermediate species, such as soluble and/or insoluble dimers, oligomers, protofibrils [7]. Therefore, oligomeric α-syn is widely used to describe aggregated α-syn [8].

In the α-syn aggregation process, post-translational modifications such as phosphorylation, ubiquitination, truncation, and oxidation by tyrosine nitration have emerged as major markers of α-syn pathology [9]. The phosphorylation of α-syn on multiple sites at the C-terminal end (S89, S129, Y125, Y133, and Y136) has important implications; phosphorylation at Serine 129 (p-S129) increases the appearance of eosinophilic cytoplasmic inclusions resembling the LBs of PD [10]. Studies have reported that approximately 90% of α-syn in LBs is phosphorylated at S129 in postmortem PD samples [11,12]. The abnormally accumulated α-syn oligomers at the presynaptic membrane in diseased neurons propagate to nearby healthy neurons via presynaptic terminals [13]. This transmission of α-syn induces mitochondrial dysfunction and promotes mitochondrial susceptibility to oxidative stress, dopamine transporter-mediated toxicity, caspase activation and cell death, which promotes the pathogenesis of PD and DLB [14,15].

Considering the above information, it is crucial to develop treatments that can reduce α-syn aggregation and impede PD progression. Interestingly, cell-based therapies with mesenchymal stem cells (MSCs) and their secretomes have been shown to cross the BBB, which explains their use in the treatment of NDDs [16]. MSCs are self-renewing and multipotent progenitor cells, and their beneficial effects against NDDs were mainly due to the factors they secrete, rather than cellular engraftment in models of PD [17,18], demonstrating the neuroprotective ability of MSC-conditioned medium (MSCs-CM). Moreover, adipose tissues represent an emerging source of stem cells; these tissues are obtained by less invasive methods such as lipoaspiration. Human adipose tissue-derived stem cells (hADSCs) differentiate into neuron- or glia-like cells in vitro [19]. Consistent with the results of the above studies, our group previously reported that neural-induced hADSCs (NI-hADSCs) displayed the functional characteristics of neuronal cells in the presence of basic fibroblast growth factor (bFGF) and forskolin for over two weeks [20]. In the present study, we evaluated the therapeutic role of NI-hADSC conditioned medium (NI-hADSC-CM) in ROT-induced PD-like impairments. We evaluated the differential alterations in the levels of soluble/insoluble monomeric, dimeric, and oligomeric forms of p-S129 and total α-syn expression. Moreover, we studied the involvement of specific cellular signaling pathways in human SH-SY5Y cells to gain valuable insights into PD pathogenesis.

## 2. Results

### 2.1. Effects of NI-hADSC-CM on ROT-Induced Cell Death in SH-SY5Y Cells

**Figure 1:**
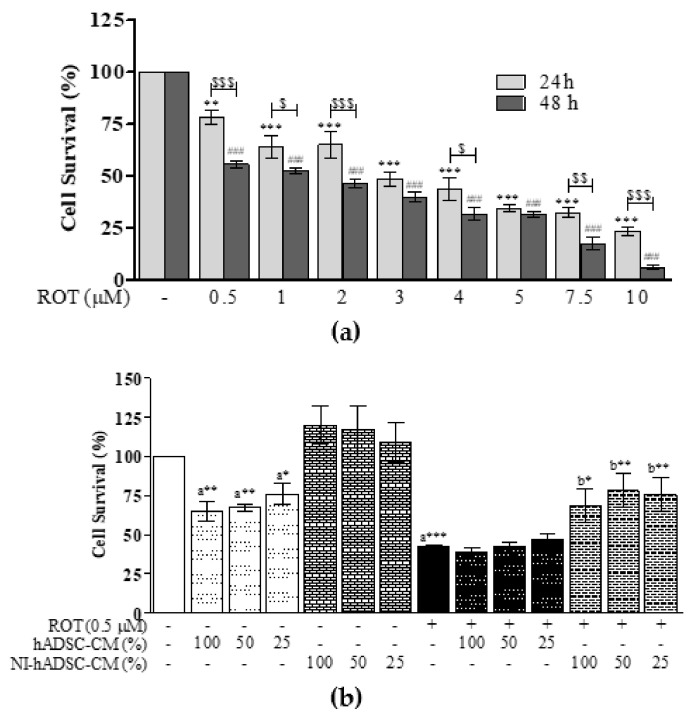
Effects of NI-hADSC-CM on ROT-induced cell death. SH-SY5Y cells were seeded at a density of 5×10^4^ cells/mL in DMEM containing 1% FBS and used for experiments after overnight incubation. (**a**) Cells were incubated with different concentrations of ROT (0, 0.5, 1, 2, 3, 4, 5, 7.5, and 10 μM) for 24 h or 48 h and subjected to trypan blue cell viability assay. Data are presented as the mean ± SEM of three independent experiments and analyzed by one-way analysis of variance (ANOVA) followed by Tukey’s post hoc test. Statistical significance: ** *p* < 0.01 and *** *p* < 0.001 vs. control for 24 h; ### *p* < 0.001 vs. control for 48 h. A two-way ANOVA followed by a Bonferroni post hoc test analyzed the time-dependent effects of ROT. Statistical significance: $ *p* < 0.05, $$ *p* < 0.01, and $$$ *p* < 0.001. (**b**) Cells were incubated in the absence or presence of ROT (0.5 μM) for 48 h and then treated with hADSC-CM or NI-hADSC-CM at 100 or 50 or 25% during the last 24 h, and cell survival was assessed by trypan blue assay. Data are presented as the mean ± SEM of three independent experiments. Statistical analysis was performed using one-way analysis of variance (ANOVA) followed by Tukey’s post hoc test. Statistical significance: a—compared with control; b—compared with ROT; * *p* < 0.05, ** *p* < 0.01, and *** *p* < 0.001.

To evaluate the toxicity of ROT on SH-SY5Y cells, dose- and time-dependent studies were performed. Cells cultured in DMEM with 1% FBS were incubated with different concentrations of ROT (0, 0.5, 1, 2, 3, 4, 5, 7.5, and 10 μM) for 24 or 48 h. Adherent cells after trypsinization and deplated floating cells were used for trypan blue cell viability assays. The cell survival rate gradually decreased with increasing concentrations of ROT, indicating that ROT induced cell death after 24 and 48 h in a dose- and time-dependent manner (Figure 1a). Based on this experiment, 0.5 μM ROT, which reduced cell viability to around 55% compared with the control group, was used in all subsequent experiments.

To test the therapeutic effects of NI-hADSC-CM, SH-SY5Y cells were first treated with or without ROT for 24 h. After removal of culture medium, cells were treated with or without hADSC-CM or NI-hADSC-CM at 100, 50, and 25% dilution in DMEM supplemented with 1% FBS and incubated in the absence or presence of ROT (0.5 μM) for another 24 h (Figure 1b). Treatment with NI-hADSC-CM at 100, 50, and 25% dilutions significantly increased the numbers of ROT-exposed cells, but the normal number of cells was maintained in case of the control groups. In contrast, ROT-exposed cells treated with hADSC-CM did not show any significant changes against ROT-induced toxicity. However, they showed a significant decrease in cell numbers compared with the normal control group. These results evidence that NI-hADSC-CM must have higher therapeutic effects compared to hADSC-CM, which showed toxicity to control SH-SY5Y cells and no significant protective effect against ROT toxicity. Morphological changes showed that ROT exposure retracted cell neurites, altered the cell surface, and reduced the cell number, compared with the control group. NI-hADSC-CM treatment increased the cell number, along with an increase in the amount of cell neurites (Supplementary Figure S1). From these results, we chose NI-hADSC-CM at 50% dilution for further experiments (Supplementary Figure S2a) and morphological observation observed (Supplementary Figure S2b).

### 2.2. Effects of NI-hADSC-CM against ROT on TH and Syn211 Protein Expressions in SH-SY5Y Cells

**Figure 2:**
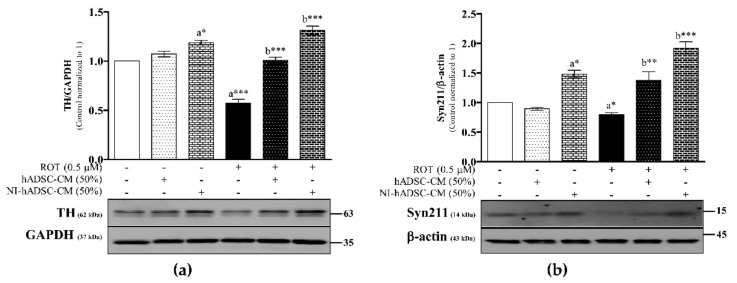
Effects of NI-hADSC-CM against ROT on TH and Syn211 protein expressions. SH-SY5Y cells were seeded at a density of 5 × 10^4^ cells/mL in DMEM containing 1% FBS and used for the experiments after overnight incubation. Cells incubated in the absence or presence of ROT (0.5 μM) for 48 h were treated with hADSC-CM (50%) or NI-hADSC-CM (50%) during the last 24 h, and the TH (**a**), Syn211 (**b**), and GAPDH or β-actin expression levels were assessed by Western blotting. Images are representative of three independent experiments. Data are presented as the mean ± SEM of three independent experiments. Statistical analysis was performed using one-way analysis of variance (ANOVA) followed by Tukey’s post hoc test. Statistical significance: a—compared with control; b—compared with ROT; * *p* < 0.05, ** *p* < 0.01, and *** *p* < 0.001.

TH, which is the rate-limiting enzyme for the biosynthesis of dopamine (DA), was evaluated by Western blotting (Figure 2a and Supplementary Figure S5). As expected, the protein expression of TH was significantly decreased following ROT toxicity (*p*<0.001) after 48 h, suggesting that ROT-induced toxicity led to neurodegeneration in SH-SY5Y cells. However, treatment with NI-hADSC-CM showed a marked protective effect (*p* < 0.001) against ROT toxicity in the last 24 h of the 48-h incubation period. hADSC-CM (50%) also showed protective effects, similar to NI-hADSC-CM.

To determine whether the expression of α-syn has been linked to PD, we used α-syn clone Syn211 antibody in Western blotting. To our surprise, results revealed that ROT-induced a significant decrease in total α-syn detected as Syn211 monomer (14 kDa), furthermore, the level of Syn211 was found to be increased in the NI-hADSC-CM or hADSC-CM treatment against ROT toxicity on SH-SY5Y cells (Figure 2b and Supplementary Figure S5).

### 2.3. Effects of NI-hADSC-CM against ROT on p-S129 and Total α-syn Protein Expressions in SH-SY5Y Cells

**Figure 3:**
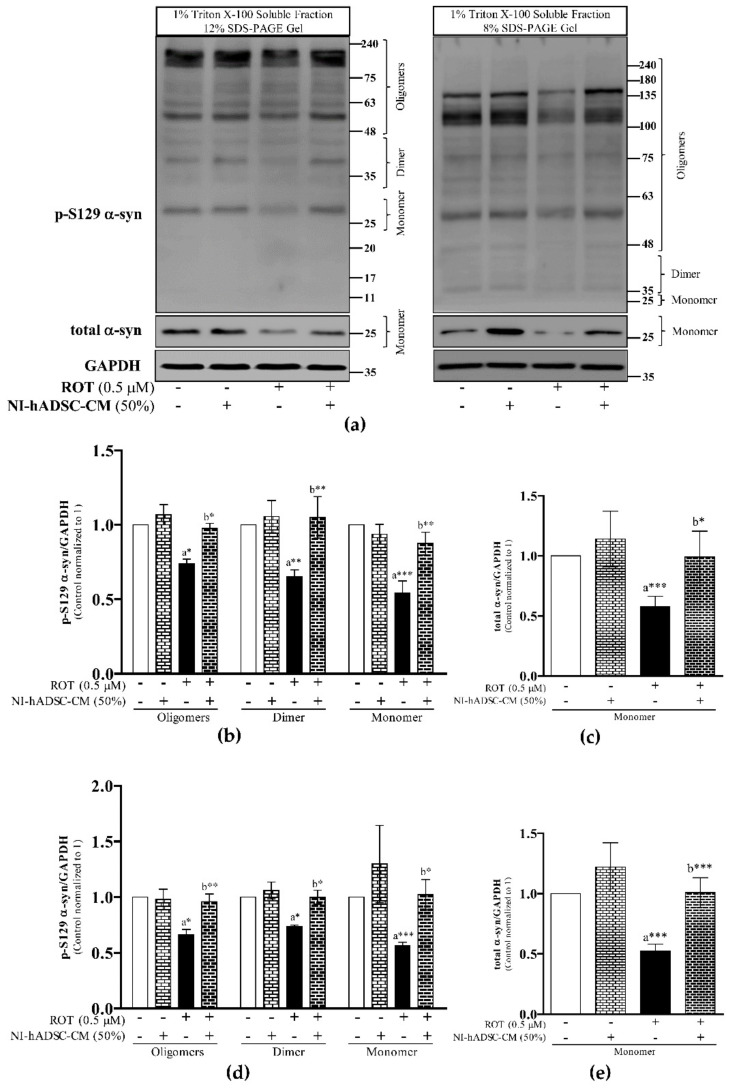
Effects of NI-hADSC-CM against ROT on p-S129 and total α-syn expressions in Triton X-100-soluble cell lysate fractions. SH-SY5Y cells were seeded at a density of 5 × 10^4^ cells/mL in DMEM containing 1% FBS and used for the experiments after overnight incubation. Cells incubated in the absence or presence of ROT (0.5 μM) for 48 h were treated with NI-hADSC-CM (50%) during the last 24 h. Cell lysates were prepared as 1% Triton X-100 soluble and insoluble (2× SDS-soluble) fractions. The expression levels of p-S129- and total α-syn were analyzed from 1% Triton X-100 soluble fractions by Western blotting using 12 and 8% SDS-PAGE gels (**a**). Images are representative of three independent experiments. The bar graphs represent fold changes in p-S129 α-syn/GAPDH (**b**,**d**) and total α-syn/GAPDH (**c**,**e**) in 12% (**b**,**c**) or 8% (**d**,**e**) SDS-PAGE. Data are presented as the mean ± SEM of three independent experiments. Statistical analysis was performed using one-way analysis of variance (ANOVA) followed by Tukey’s post hoc test. Statistical significance: a—compared with control; b—compared with ROT; * *p* < 0.01, ** *p* < 0.05, and *** *p* < 0.001.

**Figure 4:**
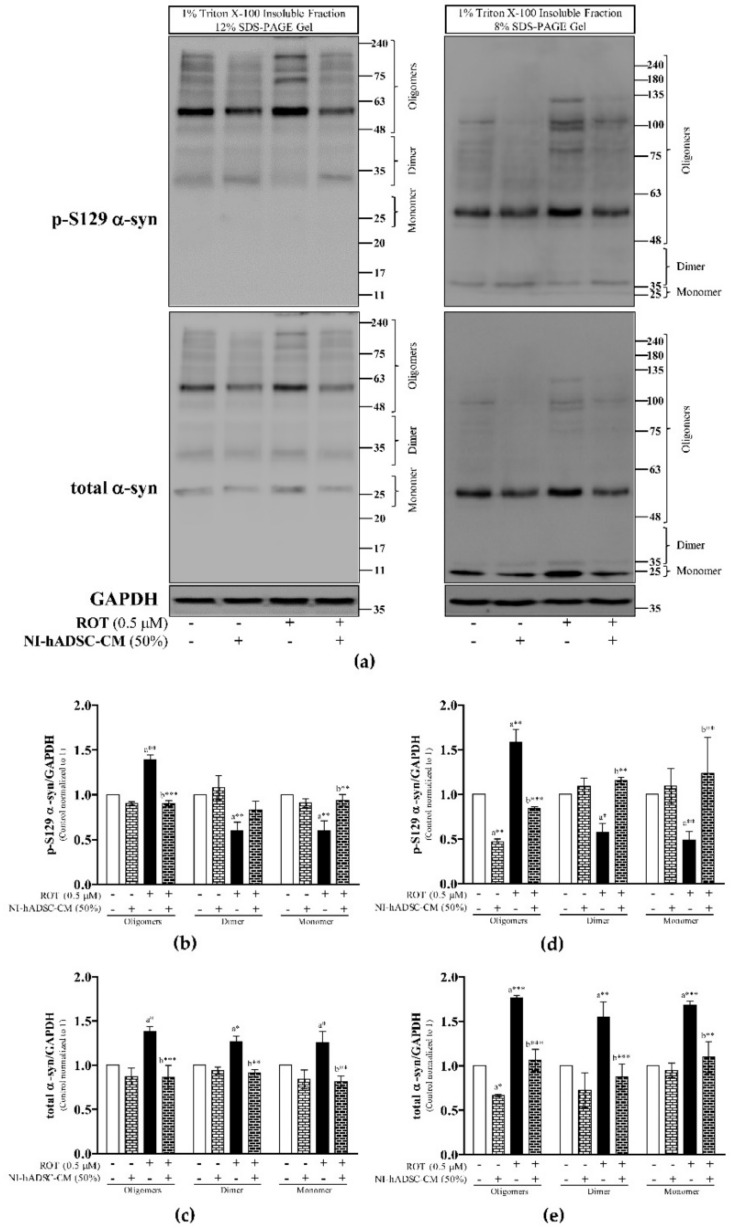
Effects of NI-hADSC-CM against ROT on p-S129 and total α-syn expressions in Triton X-100-insoluble cell lysate fractions. SH-SY5Y cells were seeded at a density of 5 × 10^4^ cells/mL in DMEM containing 1% FBS and used for experiments after overnight incubation. Cells incubated in the absence or presence of ROT (0.5 μM) for 48 h were treated with NI-hADSC-CM (50%) during the last 24 h. Cell lysates were prepared as 1% Triton X-100 soluble and insoluble (2× SDS-soluble) fractions. The expression levels of p-S129- and total α-syn were analyzed from 1% Triton X-100 insoluble (2× SDS soluble) fractions by Western blotting using 12 and 8% SDS-PAGE gels (**a**). Images are representative of three independent experiments. The bar graphs represent fold changes in p-S129 α-syn/GAPDH (**b**,**d**) and total α-syn/GAPDH (**c**,**e**) in 12% (**b**,**c**) or 8% (**e**) SDS-PAGE gels. Data are presented as the mean ± SEM of three independent experiments. Statistical analysis was performed using one-way analysis of variance (ANOVA) followed by Tukey’s post hoc test. Statistical significance: a—compared with control; b—compared with ROT; * *p* < 0.05, ** *p* < 0.01, and *** *p* < 0.001.

The crucial neuropathological feature in PD is the progressive accumulation of α-syn aggregates. To assess the molecular underpinnings of ROT-induced α-syn aggregation in SH-SY5Y cells, Western blot analyses of the Triton X-100-soluble and -insoluble (2% SDS soluble) lysate fractions were performed (Figure 3 and Figure 4). hADSC-CM, which showed toxicity to control SH-SY5Y cells on cell viability study (Figure 1b), but showed significant protective effect on TH and Syn211 against ROT toxicity (Figure 2) might not suitable to use against NDDs, therefore, hADSC-CM was omitted here. The Triton X-100-soluble and -insoluble (2% SDS soluble) protein fractions from 4 groups (depicted in Supplementary Figure S3a) were loaded onto 12 and 8% SDS-PAGE gels and immunoblotted for detecting the oligomeric, dimeric, and monomeric forms of p-S129 and total α-syn protein expression levels (Figure 3 and Figure 4).

The analysis of the Triton X-100-soluble fraction (Figure 3a and Supplementary Figure S6) revealed that 48 h of 0.5 μM ROT toxicity significantly decreased the levels of the oligomeric (*p* < 0.05 with 12 and 8% SDS-PAGE), dimeric (*p* < 0.01 and *p* < 0.05 with 12 and 8% SDS-PAGE, respectively), and monomeric (*p* < 0.001 with 12 and 8% SDS-PAGE) forms of p-S129 α-syn as well as the total monomeric α-syn levels (*p* < 0.001 with 12 and 8% SDS-PAGE) (Figure 3b–e).

However, the levels of p-S129 and total α-syn were significantly increased after treatment with NI-hADSC-CM for the last 24 h of the 48-h incubation period, compared to ROT exposure alone. The levels of the oligomeric (*p* < 0.05 and *p* < 0.01 with 12 and 8%, respectively), and dimeric and monomeric (*p* < 0.01 and *p* < 0.05 with 12 and 8% SDS-PAGE, respectively) forms of p-S129 α-syn as well as total monomeric α-syn (*p* < 0.05 and *p* < 0.05 with 12 and 8% SDS-PAGE, respectively), increased as seen in Figure 3b–e. Additionally, the ratio of monomeric p-S129/total α-syn was not changed following ROT-induced toxicity and NI-hADSC-CM treatment (Supplementary Figure S3b,c; 12 and 8% SDS-PAGE, respectively), suggesting that the expression of p-S129 was positively correlated with that of total α-syn.

As shown in Figure 4 and Supplementary Figure S7, we compared the Triton X-100-insoluble p-S129 protein expression levels with the total α-syn expression levels.

We observed that ROT toxicity induced an increase in the levels of oligomeric (p < 0.01 with 12 and 8% SDS-PAGE) p-S129 α-syn, but a decrease in the expression levels of dimeric (*p* < 0.01 and *p* < 0.05 with 12 and 8% SDS-PAGE, respectively) and monomeric (*p*<0.01 with 12 and 8% SDS-PAGE) p-S129 α-syn (Figure 4b,d, respectively from 12 and 8% SDS-PAGE). NI-hADSC-CM treatment significantly reversed the effects of ROT on the oligomeric (*p* < 0.001 with 12 and 8% SDS-PAGE), dimeric (*p* > 0.05 and *p* < 0.01 with 12 and 8% SDS-PAGE, respectively), and monomeric (*p* < 0.01 with 12 and 8% SDS-PAGE) p-S129 protein expression levels.

Moreover, the oligomeric, dimeric, and monomeric forms (12% SDS-PAGE: *p* < 0.05; 8% SDS-PAGE: oligomeric and monomeric α-syn at *p* < 0.001, dimeric α-syn at *p* < 0.01) of total α-syn in the insoluble fractions were significantly increased following ROT-induced toxicity, compared with the corresponding levels in the control samples. Treatment of ROT-exposed cells with NI-hADSC-CM for the last 24 h of the 48-h incubation period reduced the expression levels of the oligomeric (*p* < 0.001 with 12 and 8% SDS-PAGE), dimeric (*p* < 0.01 and *p* < 0.001 with 12 and 8% SDS-PAGE, respectively gel), and monomeric (*p* < 0.01 with 12 and 8% SDS-PAGE) forms of total α-syn to approximately the levels observed in the control samples (Figure 4c and 4e, respectively from 12 and 8% SDS-PAGE). In addition, the ratio of oligomeric p-S129/total α-syn in ROT-exposed cells treated with NI-hADSC-CM did not change (Supplementary Figure S3d,e), suggesting that the expression levels of insoluble oligomers of p-S129 were positively correlated with those of oligomeric total α-syn. The ratio of dimeric and monomeric p-S129/total α-syn following ROT-induced toxicity was decreased, but that in NI-hADSC-CM-treated cells were increased to the levels observed in the control samples (Supplementary Figure S3d,e; 12 and 8% SDS-PAGE, respectively). The hADSC-CM treatment, which showed toxicity to control SH-SY5Y cells and no protective effect against ROT-induced toxicity was not used in Western blot to detect p-S129 and/or total α-syn.

### 2.4. Effects of NI-hADSC-CM against ROT on Protein Expression of Neuronal Markers in SH-SY5Y Cells

**Figure 5:**
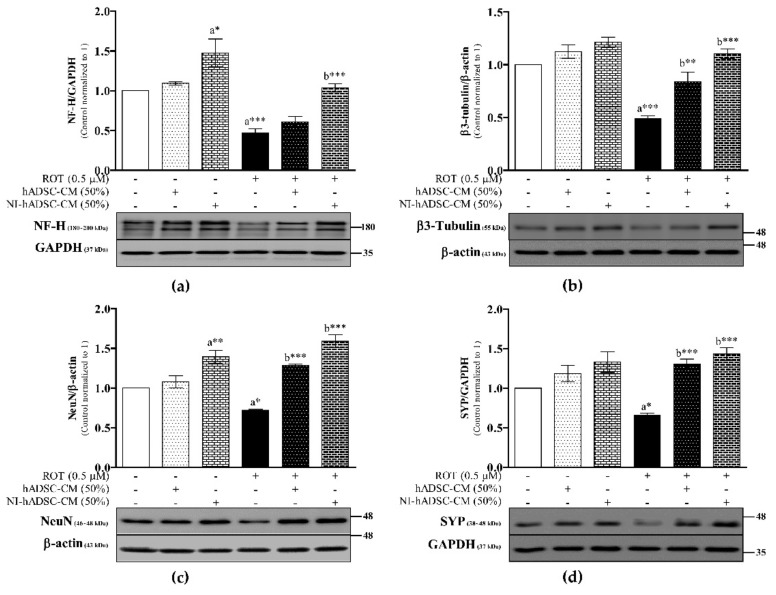
Effects of NI-hADSC-CM against ROT on protein expression of neuronal markers. SH-SY5Y cells were seeded at a density of 5 × 10^4^ cells/mL in DMEM containing 1% FBS and used for experiments after overnight incubation. Cells in the absence or presence of ROT (0.5 μM) for 48 h were treated with hADSC-CM (50%) or NI-hADSC-CM (50%) during the last 24 h, and the expression levels of NF-H (**a**), β3-tubulin (**b**), NeuN (**c**), SYP (**d**), and GAPDH or β-actin were analyzed by Western blotting. Images are representative of three independent experiments. Data are presented as mean ± SEM of three independent experiments. Statistical analysis was performed using one-way analysis of variance (ANOVA) followed by Tukey’s post hoc test. Statistical significance: a—compared with control; b—compared with ROT; * *p* < 0.05, ** *p* < 0.01, and *** *p* < 0.001.

Phosphorylated α-syn can interact with several cytoskeletal and synaptic proteins and affect their neuronal functions. To evaluate this aspect, we investigated the protein expression of the neuronal markers neurofilament-heavy (NF-H), β3-tubulin (Tuj1), neuronal nuclei (NeuN), and synaptophysin (SYP) (Figure 5 and Supplementary Figure S8). ROT-exposed cells showed a significant decrease in the expression levels of NF-H (*p* < 0.001; Figure 5a), β3-tubulin (*p* < 0.001; Figure 5b), NeuN (*p* < 0.05; Figure 5c), and SYP (*p* < 0.05; Figure 5d) compared with the control group. As expected, the NF-H, β3-tubulin, NeuN, and SYP expression levels were increased by NI-hADSC-CM (*p* < 0.001 for all) and hADSC-CM (β3-tubulin: *p* < 0.01; NeuN and SYP: *p* < 0.001) in ROT-exposed SH-SY5Y cells. Treatment of control cells with NI-hADSC-CM increased the expression levels of NF-H (*p* < 0.05) and NeuN (*p* < 0.01).

### 2.5. Effects of NI-hADSC-CM against ROT on Apoptotic Protein Expression Levels in SH-SY5Y Cells

**Figure 6:**
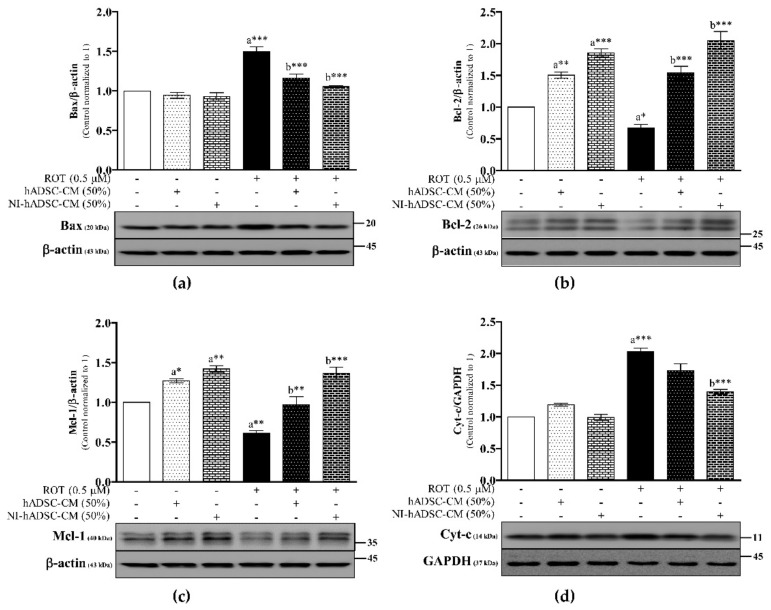
Effects of NI-hADSC-CM against ROT on Bcl-2 family proteins and Cyt-c expressions. SH-SY5Y cells were seeded at a density of 5 × 10^4^ cells/mL in DMEM containing 1% FBS and used for experiments after overnight incubation. Cells in the absence or presence of ROT (0.5 μM) for 48 h were treated with hADSC-CM (50%) or NI-hADSC-CM (50%) during the last 24 h and the Bax (**a**), Bcl-2 (**b**), Mcl-1 (**c**), Cyt-c (**d**) and β-actin or GAPDH expression levels were assessed by Western blotting. Images are representative of three independent experiments. Data are presented as mean ± SEM of three independent experiments. Statistical analysis was performed using one-way analysis of variance (ANOVA) followed by Tukey’s post hoc test. Statistical significance: a—compared with control; b—compared with ROT; * *p* < 0.05, ** *p* < 0.01, and *** *p* < 0.001.

As the abnormal accumulation of α-syn oligomers during oxidative stress leads to mitochondrial dysfunction, we detected the levels of the pro-apoptotic protein Bax and anti-apoptotic proteins Bcl-2 and Mcl-1 to examine the effects of NI-hADSC-CM on the expression of apoptosis-related proteins in ROT-exposed SH-SY5Y cells (Figure 6 and Supplementary Figure S9). We found that ROT exposure for 48 h significantly increased the levels of Bax (*p* < 0.001; Figure 6a) but decreased those of Bcl-2 (*p* < 0.05; Figure 6b) and Mcl-1 (*p* < 0.01; Figure 6c), compared with the control group. However, treatment with NI-hADSC-CM or hADSC-CM significantly decreased the Bax levels (*p* < 0.001) and increased the Bcl-2 (*p* < 0.001) and Mcl-1 (*p* < 0.001 by NI-hADSC-CM; *p* < 0.01 by hADSC-CM) levels in ROT-exposed SH-SY5Y cells. Moreover, control cells treated with NI-hADSC-CM or hADSC-CM showed increased Bcl-2 (*p* < 0.001 by NI-hADSC-CM; *p* < 0.01 by hADSC-CM) and Mcl-1 (*p* < 0.01 by NI-hADSC-CM; *p* < 0.05 by hADSC-CM) protein expression levels compared to those in the untreated control SH-SY5Y cells. Bcl-2, as an anti-apoptotic member of the Bcl-2 family, can bind to Bax to form Bcl-2:Bax heterodimers, thereby attenuating the apoptotic effect of Bax. Apparently, ROT caused an increase in the Bax/Bcl-2 ratio (*p* < 0.001; Supplementary Figure S4a) but decreased the Bcl-2/Bax ratio (*p* < 0.001; Supplementary Figure S4b). Treatment with NI-hADSC-CM or hADSC-CM significantly decreased the Bax/Bcl-2 ratio (*p* < 0.001; Supplementary Figure S4a) but increased the Bcl-2/Bax ratio (*p* < 0.001; Supplementary Figure S4b), compared with the ROT-exposed group as well as the untreated control group.

Cytochrome c (Cyt-c), an activator of Cas-9, released from mitochondria to cytosol during apoptosis by ROT after overexpression of Bax levels. In Figure 6d, SH-SY5Y cells with ROT toxicity led a significant increase in Cyt-c protein expression (*p* < 0.001) compared with the control group; however, NI-hADSC-CM treatment significantly attenuated the excessive expression of Cyt-c (*p* < 0.001). Treatment of hADSC-CM to ROT-induced SH-SY5Y cells did not show any significant changes on Cyt-c levels.

**Figure 7:**
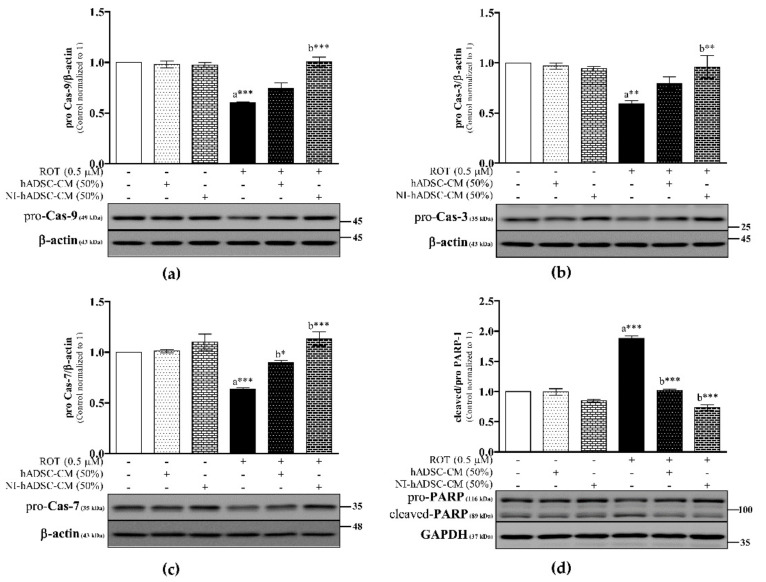
Effects of NI-hADSC-CM against ROT on caspases and PARP-1 protein expressions. SH-SY5Y cells were seeded at a density of 5 × 10^4^ cells/mL in DMEM containing 1% FBS and used for experiments after overnight incubation. Cells in the absence or presence of ROT (0.5 μM) for 48 h were treated with hADSC-CM (50%) or NI-hADSC-CM (50%) during the last 24 h, and the expression levels of pro-Cas-9 (**a**), pro-Cas-3 (**b**), pro-Cas-7 (**c**), PARP (**d**), and β-actin or GAPDH were assessed by Western blotting. Images are representative of three independent experiments. Data are presented as the mean ± SEM of three independent experiments. Statistical analysis was performed using one-way analysis of variance (ANOVA) followed by Tukey’s post hoc test. Statistical significance: a—compared with control; b—compared with ROT; * *p* < 0.05, ** *p* < 0.01, and *** *p* < 0.001.

As shown in Figure 7 and Supplementary Figure S10, the levels of pro-Cas-9, -3, and -7 were significantly downregulated (*p* < 0.001, *p* < 0.01, and *p* < 0.001, respectively), similar to the case for the Bcl-2 and Mcl-1 expression levels in the ROT-induced toxicity group. Caspases are synthesized as inactive pro-caspases undergo cleavage leading to their activation. Unfortunately, we did not get any cleaved signals in Western blotting. The decreased pro-caspases during ROT-induced toxicity have been evidence the increased cleaved/active caspases. Treatment with NI-hADSC-CM significantly increased the levels of pro-caspases (Cas-9 and Cas-7: *p* < 0.001; Cas-3: *p* < 0.01) in the ROT-exposed cells. hADSC-CM treatment also increased the pro-Cas-7 (*p* < 0.05) levels following ROT toxicity. Moreover, the levels of pro-PARP-1 decreased (*p* < 0.05; Supplementary Figure S4b), but those of cleaved PARP-1 (*p* < 0.05; Supplementary Figure S4c) and the cleaved/pro-PARP-1 ratio (*p* < 0.001; Figure 7d) increased following ROT-induced toxicity in SH-SY5Y cells. NI-hADSC-CM treatment increased the pro-PARP-1 (*p* < 0.05) levels and decreased the cleaved PARP-1 levels (*p* < 0.01) and cleaved/pro-PARP-1 ratio (*p* < 0.001; Figure 7d) following ROT-induced toxicity. hADSC-CM treatment decreased the cleaved PARP-1 levels (*p* < 0.05) and the cleaved/pro-PARP-1 ratio (*p* < 0.001) following ROT-induced toxicity in SH-SY5Y cells. These results demonstrated that NI-hADSC-CM could inhibit the ROT-induced apoptosis in SH-SY5Y cells.

## 3. Discussion

MSCs are unspecialized cells in the human body that can be differentiated into specialized stem cells for performing diverse functions. This property of MSCs has been exploited for the treatment of various diseases [21]. We previously established a protocol for the isolation and culture of hADSCs. More than 95% of the hADSCs expressed MSC-specific markers, including CD13, CD44 (endoglin), CD90 (Thy-1), and CD166, but did not express markers for hematopoietic stem cells, including CD14, CD34, and CD45 [20]. After neurogenic differentiation in the presence of bFGF and forskolin for over two weeks, the majority of NI-hADSCs exhibited distinct bipolar or multipolar morphologies with branched processes. The expression of neural stem cell marker (nestin), neuronal markers (Tuj1, MAP2, NF-L, NF-M, NF-H, NSE, and NeuN), synaptic markers (GAP43 and SNAP25), astrocyte marker (GFAP), and oligodendrocyte marker (CNPase) was very high when NI-hADSCs were grown in the presence of bFGF and forskolin. They also displayed voltage-dependent and TTX-sensitive sodium currents, which are functional hallmarks of neurons, and expressed high levels of ionic channel genes, which are important in neural function [20]. Moreover, our previous study showed that the transplantation of NI-hADSCs could restore the injured spiral ganglion neurons in guinea pigs with neomycin-induced sensorineural hearing loss [22]. We also previously reported that neural-induced human bone-marrow stem cells (NI-hBMSCs) by bFGF and forskolin markedly increased the expression of neurotrophic factors (NGF, bFGF, angiopoietin-1, BDNF, GDNF and NT-3) after transplanted to guinea pig model for facial nerve axotomy injury. These results suggests that NI-hBMSCs act as a source of neurotrophic factors [23].

Secretome or conditioned medium (CM) contains a set of bioactive factors/molecules released from cells, tissues, or organisms [24,25]. These factors include secreted lipids, proteins, nucleic acids, chemokines, cytokines, growth factors, hormones, and extracellular vesicles (EVs) [26,27]. Treatment using MSC-CM conferred effective therapeutic benefits against NDDs, including focal cerebral ischemia-reperfusion injury, by inducing the recovery of damaged cells and tissues [28]. In the present study, the tested concentrations of ROT caused dose- and time-dependent reductions in cell survival. However, treatment with NI-hADSC-CM for the last 24 h markedly attenuated the ROT (0.5 μM)-induced toxicity for 48 h. Moreover, hADSC-CM showed toxicity to SH-SY5Y cells and no significant protective effect against ROT toxicity might not be suitable to use against NDDs, therefore, the beneficial effects of hADSC-CM on cellular signaling was not discussed here.

TH is an important rate-limiting enzyme in dopamine biosynthesis as well as in physiological brain functions [2]. ROT-induced toxicity for 48 h dramatically reduced the expression of TH, suggesting that the PD-like impairments in SH-SY5Y cells correlate with the magnitude of dopamine deficit that impairs dopamine synthesis and its metabolism in neurons undergoing selective degeneration [2,3]. Meanwhile, the upregulation of TH expression by NI-hADSC-CM treatment in ROT-exposed cells indicates the enhanced dopamine production and diminished ROT-induced PD-like impairments.

The reduction of TH expression in PD may indicate changes in the biosynthesis of proteins required for axonal regeneration in neurotransmission [29]. The localization of α-syn on synaptic vesicles as well as in cell bodies and axons of neuronal cells [30] has several important regulatory functions, including synaptic maintenance, mitochondrial homeostasis, proteasome function, dopamine metabolism, and chaperone activity [31]. α-syn is also the main component of LBs in the pathogenesis of PD. α-syn clone Syn211 which can only bind once per α-syn C-terminus region residues 121–125 [32] decreased during ROT toxicity indicates a significant impairment in the presynaptic neurotransmission. This was evidenced by others that the knock-down of α-syn causes neuronal dysfunction and degeneration [33], reduction in striatal dopamine in mice [34], and impaired synaptic vesicle docking and the release of neurotransmitters into the synaptic cleft in traumatic brain injury in rats [35]. The level of Syn211 was found to be increased in the NI-hADSC-CM group shown that soluble monomeric α-syn is essential for α-syn-related neurotransmission. In addition, these results indicate that further experiments are needed to delineate the exact soluble and aggregated α-syn in the context of NDDs.

Decreased salivary total a-syn may reflect the reduction of α-syn monomers as well as the formation of insoluble intracellular inclusions and soluble oligomers in PD patients [36]. The excess α-syn aggregation in the dopaminergic neuronal terminals also reduces the dopamine reuptake [37]. Natively unfolded α-syn monomers interact to form unstable dimers, which develop into toxic oligomers and fibrils in PD [38,39]. These toxic species of α-syn may propagate from diseased cells to healthy neurons and induce the conversion of native α-syn into toxic oligomeric species [40]. The phosphorylation of α-syn at Serine129 (p-S129) in the C-terminus (95–140) leads to a higher propensity for α-syn aggregation [41]. Moreover, p-S129 is implicated in the formation of LBs and α-syn toxicity [42]. Therefore, the expression levels of p-S129 and the total oligomeric, dimeric, and monomeric forms of α-syn in Triton X-100-soluble and -insoluble (2% SDS soluble) fractions were analyzed by Western blotting using paraformaldehyde and glutaraldehyde as membrane fixatives.

As anticipated, the level of the oligomeric form of insoluble p-S129 α-syn was increased in proportion to that of the insoluble total α-syn oligomeric protein during ROT toxicity along with a reduced level of total α-syn soluble oligomers, indicating that ROT increased the toxic α-syn aggregation with PD disease progression. Previous studies have suggested that increased p-S129 α-syn levels in PD patients leads to α-syn aggregation [43,44]. Other studies have provided evidence that the oligomeric α-syn is positively correlated with the motor impairments in PD patients [45,46,47] and aged mice [48]. In addition, the oligomerization of α-syn could be initiated by dimerization from monomeric α-syn on membrane surfaces [49]. The levels of insoluble dimeric and monomeric p-S129 were decreased following ROT-induced toxicity, in contrast with the increased levels of dimeric and monomeric forms of total α-syn, suggesting that insoluble p-S129 α-syn is mostly associated with oligomeric α-syn aggregation rather than dimeric or monomeric α-syn aggregation. Moreover, the increased levels of the monomeric and dimeric forms of total α-syn observed in the Triton X-100-insoluble fraction of ROT-exposed cells in this study indicates that ROT-induced inhibition of mitochondrial complex I activity affects the stability of monomeric α-syn [50] and is involved in the initiation and the accumulation of the oligomeric forms of total α-syn [51]. In addition, the unchanged ratio of soluble monomeric p-S129/total α-syn levels during ROT-induced toxicity suggests that the expression of soluble p-S129 was also positively correlated with that of total soluble α-syn.

In our study, we found that NI-hADSC-CM treatment significantly reduced the accumulation of insoluble p-S129 oligomers and total α-syn levels, but increased the soluble p-S129 and total α-syn levels during ROT-induced toxicity. A previous study has shown that MSC-CM treatment reduced the formation of α-syn aggregates [52], suggesting that the insoluble p-S129 and total α-syn oligomers in our study were converted into soluble oligomers possibly due to the enhanced clearance of α-syn aggregates by NI-hADSC-CM treatment. NI-hADSC-CM likely preserved the levels of the soluble monomeric form of the α-syn protein and its physiological functions. Therefore, the inhibitory effect of NI-hADSC-CM on the levels of α-syn oligomers might be due to its active constituents, which penetrate the BBB to ameliorate the ROT-induced impairments, as previously reported [53]. We hypothesize that the reduction of the levels of insoluble oligomeric p-S129 and total α-syn, along with the preservation of the levels of soluble α-syn, may be sensitive markers for the treatment of LBs in PD.

ROT-induced inhibition of the activity of mitochondrial complex I by oxidative stress ultimately impaired mitochondrial function via disturbed mitochondrial dynamics subsequently damages the cytoskeletal proteins [54], interferes with the production of synaptic vesicle proteins [55], and destroys the axonal transport integrity [56], leading to neurotransmitter leakage [57]. Neurofilaments (NFs) are intermediate filamentary proteins that play a role in forming and maintaining the axonal architecture and transport cargoes within the neurons [58]. NF-H (also called NF200) is a major component of axonal outgrowth [59] with a more mature phenotype [60]. The decreased expression of NF-H in this study is consistent with previous findings that the overexpression of α-syn affects the integrity of neurofilament networks [61] and leads to motor neurons retracting from synapses [62]. NI-hADSC-CM treatment promoted NF-H protein expression, suggesting that the integrity of the neuronal cytoskeleton is necessary for axonal growth and cargo transport.

Overexpression of α-syn oligomers is associated with the disruption of neurofilament networks, microtubule structures, and axonal transport in normal neurons [63]. β3-tubulin (Tuj1, also called TUBB3) is a microtubule-related neuronal cell marker expressed exclusively in neurons [64]. Moreover, NF-H directly binds to the C-terminal domain of tubulin and modulates its activity [65]. In the present study, β3-tubulin expression was significantly reduced during ROT toxicity, which is consistent with a previous study that β3-tubulin levels were dramatically decreased in NDDs [66]. It was hypothesized that the polymerization of β3-tubulin with α-syn forms an insoluble protein complex that accumulates in the nerve terminals leading to neuronal dysfunction, indicating that microtubules play an important role in α-syn regulation [67]. ROT-exposed cells showed increased β3-tubulin and soluble α-syn levels after NI-hADSC-CM treatment, suggesting that the expression of β3-tubulin is essential for progressive neurite formation and growth.

Neuronal nuclear protein (NeuN, also called Fox-3 or RBFOX3) is a highly conserved and soluble nuclear protein that binds to DNA and is observed in most neuronal cell types throughout the nervous system. NeuN expression occurs in association with terminal neuronal differentiation [68,69]. Therefore, NeuN is used to evaluate neuronal cell loss in NDDs [70]. Given this fact, the present study also revealed a significant loss of this marker NeuN during ROT-induced toxicity. However, NI-hADSC-CM increased NeuN expression, indicating the neuronal differentiation of SH-SY5Y cells. Therefore, NeuN has been a reliable marker of mature neurons [71].

A membrane glycoprotein localized in presynaptic vesicles, SYP is associated with recycling vesicles that are essential for neurotransmission [72]. SYP expression has been used to access synaptic density in the brain [73] and cultured neurons [74]. Accumulation of α-syn in the synaptic C-terminus resulted in reduced SYP localization in the terminals [49]. In our study, the decreased expression of the SYP protein indicates the synaptic degeneration or reduced localization or expression of SYP as a result of α-syn accumulation in the synapse, as reported previously [75]. Our results also revealed that NI-hADSC-CM treatment increased the expression of SYP and decreased the levels of α-syn oligomers in the ROT-exposed SH-SY5Y cells, suggesting that NI-hADSC-CM inhibits the accumulation of α-syn oligomers in the synapses and facilitates optimal SYP function. Finally, NI-hADSC-CM treatment was correlated with the expression of the neuronal markers, confirming that NI-hADSC-CM reduced axonal injury and enhanced axonal and synaptic properties after ROT-induced toxicity.

Overexpression of α-syn or its oligomers with annular and pore-like structures in the membrane exerts a cytotoxic effect on neurons, which is linked to the apoptotic Bcl-2 family-related caspase pathways [76]. Apoptosis-induced neuronal death is the main hallmark of PD by ROT-induced toxicity, which inhibits the activity of mitochondrial ETC complex I, resulting in the generation of intracellular ROS through electron accumulation [77]. The mitochondrial permeabilization is regulated by the Bcl-2 family of proteins, which consists of several pro-apoptotic and anti-apoptotic members. The pro-apoptotic protein Bax resides in the cytosol. Bcl-2, an anti-apoptotic protein residing in the outer mitochondrial membrane, inhibits cytochrome c release [78,79], stabilizes the membrane potential, preserves ATP production, and prevents oxidative stress [80]. In this study, ROT toxicity induced a significant increase in Bax expression but decreased the expression of Bcl-2 and Mcl-1. In addition, the Bax/Bcl-2 protein ratio is a better predictor of apoptotic cell death than the absolute concentrations of either Bax or Bcl-2 alone [81], which increased during ROT toxicity in SH-SY5Y cells, indicating the increased level of mitochondrial apoptosis. As expected, NI-hADSC-CM treatment reduced the Bax/Bcl-2 ratio, along with increased Mcl-1 levels in SH-SY5Y cells, indicating the restored expression levels of the mitochondrial functional proteins [79].

ROT induces the translocation of Bax from the cytosol to the mitochondria, which triggers the release of cytochrome c (Cyt-c) from the mitochondria to the cytosol [79,82], leading to the activation of caspases, and ensures apoptosis [77]. Caspases are initially synthesized as inactive pro-caspases, which undergo dimerization or oligomerization and then, cleavage, leading to their activation [83]. Cas-9 is the initiator of the caspase cascade, which can cleave and activate Cas-3 and −7 [84]. In this study, ROT toxicity significantly increased the levels of Cyt-c, activation of the initiator Cas-9 as well as the effectors Cas-3 and −7, as evidenced by the decrease in the levels of the pro-caspases, all of which are critical mediators of mitochondria-mediated apoptosis. Cyt-c released from the mitochondria directly binds to Apaf-1 in a dATP-dependent manner and induces a conformational change, allowing Cas-9 to interact with the complex to form the apoptosome, subsequently activating Cas-9 [85]. NI-hADSC-CM treatment against ROT toxicity regulated the levels of the Cyt-c and pro-caspases, suggesting that NI-hADSC-CM inhibited the expression of intracellular apoptosis-associated proteins.

Moreover, PARP-1 signaling is also involved in NDDs, probably via the induction of mitochondrial defects [77]. PARP-1 has DNA-binding domains that detect DNA damage and facilitate repair. PARP-1 is cleaved into 89-kDa fragments during apoptosis, which could have been generated by the activation of Cas-3 and -7 [86], evidenced in this study by ROT-induced toxicity in SH-SY5Y cells. Excessive activation of caspases and PARP-1 triggers the apoptotic processes, including biochemical and morphological changes, such as chromatin condensation, nuclear fragmentation, and cytoskeletal degradation [87]. These processes result in the depletion of nicotinamide adenine dinucleotide (NAD) and ATP, leading to cellular energy failure and cell death [88]. PARP-1 inhibitors have been shown to reduce α-syn-induced cell death [89], supporting that NI-hADSC-CM treatment in this study diminished ROT-induced apoptotic cell death. Therefore, our findings indicate the strong anti-apoptotic potential of NI-hADSC-CM.

Moreover, there may be suspicion that the beneficial effects of NI-hADSC-CM results in this study may be the presence of bFGF and forskolin. To explain that the clinical application of bFGF is greatly limited by its short half-life which degrade quickly and lose its bioactivities [90]. It has been shown to be unstable at physiological conditions, with a half-life of approximately eight hours at standard mammalian cell culture conditions (37 °C/5% CO_2_) [91]. bFGF did not influence the cell viability against MPP^+^ or 6-OHDA in SH-SY5Y cells [92]. Forskolin did not change the total TH in SH-SY5Y cells [93] or α-syn-induced toxicity [94]. Forskolin (10 μM) to PC12 cells decreased the cell viability at 48 h, in addition, phosphorylated and total TH did not increase in PC12 cells, but led to apoptosis mediated by caspase-3 activation [95]. However, in this present study, the NI-hADSC-CM treatment increased the cell viability, TH, and pro-caspase-3 protein levels. Our previous study resulted that transplanted NI-hBMSCs increased the neurotrophic factors in guinea pig [23]. RT-PCR resulted that neuronal transcription factors such as Neurogenin 1, Math1, and Hash1 were significantly increased in NI-hADSCs compared with hADSCs [22]. From this, the present study results explain that the beneficial effects of NI-hADSC-CM is from its bioactive factors/molecules released from NI-hADSCs and not from the differentiation compounds such as bFGF and forskolin.

## 4. Materials and Methods

### 4.1. Preparation of hADSCs and Neurogenic Differentiation of NI-hADSC

Adipose tissues were obtained from individuals according to the guidelines established by the Ethics Committee at the Chonnam National University Medical School (IRB: I-2009-03-016). hADSCs were cultured and differentiated into NI-hADSCs following our previously established methods [20,22,96]. The hADSCs were grown as adherent cultures in Dulbecco’s Modified Eagle’s medium (DMEM; Hyclone, Logan, UT, USA) supplemented with 10% fetal bovine serum (FBS; Hyclone), 1% penicillin-streptomycin (Gibco BRL, Grand Island, NY, USA), and 0.2% amphotericin B (Gibco) at 37 °C in a humidified atmosphere of 5% CO_2_. For experiment, hADSCs (passages 3-5) were maintained in DMEM containing 1% FBS for 7 days, then, the cell culture medium (hADSC-CM) was aspirated, pooled, sterile filtered using a 0.2-μm syringe filter, and stored at −80 °C until further use. To induce neurogenic differentiation, hADSCs (passages 3-5)) were maintained in DMEM containing 1% FBS and supplemented with 100 ng/mL basic fibroblast growth factor (bFGF; Invitrogen Co., Carlsbad, CA, USA) for seven days. The cells were then incubated in the presence of 10 μM forskolin (Sigma Chemical co., St. Louis, MO, USA) for the next seven days. Then, the neural-induced conditioned medium (NI-hADSC-CM) was aspirated, pooled, sterile filtered using a 0.2-μm syringe filter, and stored at −80 °C until further use. We collected multiple batches of hADSC-CM and NI-hADSC-CM for our experiments.

### 4.2. SH-SY5Y Culture

The human neuroblastoma cell line SH-SY5Y (RRID: CVCL_0019; ATCC^®^ CRL-2266) was maintained in DMEM (Welgene Inc. Gyeongsan, South Korea) supplemented with 10% FBS and 1% penicillin-streptomycin at 37 °C in a humidified atmosphere containing 5% CO_2_/95% air. Confluent cultures (passages 15–22) were washed with phosphate-buffered saline (PBS), detached with 0.25% trypsin-EDTA solution, reseeded at a density of 5 × 10^4^ cells/mL in DMEM containing 1% FBS, and used for experiments after overnight incubation.

### 4.3. Rotenone Toxicity

ROT (Sigma R8875) stock was prepared at a concentration of 10 mM in dimethyl sulfoxide (DMSO; Sigma, D2650), aliquoted, stored at −80 °C, and used within 6 months. Before starting each experiment, a ROT working solution was prepared by diluting the stock with serum-free DMEM media. SH-SY5Y cells were incubated in the absence or presence of ROT or DMSO at the indicated concentrations for 24 or 48 h. The phase contrast images were taken using an Olympus microscope (CKX41) equipped with a camera. Damaged and deplated floating cells in the medium and trypsinized cells were combined and subjected to trypan blue cell viability assay. The number of surviving cells were counted using LUNA-II^TM^ (Logos Biosystems, Anyang, South Korea) automated cell counter. The cell count assay was performed in triplicate and expressed as a percentage (%) of the control.

### 4.4. Treatments of hADSC-CM and NI-hADSC-CM

To test the therapeutic effects of NI-hADSC-CM, SH-SY5Y cells were first treated with or without ROT for 24 h. The culture medium were removed, floating cells were pelleted from the medium, resuspend the cell pellet in the fresh medium, and added to respective wells. Then, cells were treated with or without hADSC-CM or NI-hADSC-CM at 100, 50, and 25% dilution in DMEM supplemented with 1% FBS and incubated in the absence or presence of ROT (0.5 μM) for another 24 h. FBS was maintained at a concentration of 1% throughout the study. Floating cells in the medium and trypsinized adherent cells were combined and then subjected to cell counting as explained above.

### 4.5. Preparation of Total Cell Lysates and Immunoblotting

SH-SY5Y cells were incubated in the absence or presence of ROT (0.5 μM) or the solvent (DMSO) for 24 h. After removing the medium, cells were treated with or without hADSC-CM or NI-hADSC-CM at 50% dilution in DMEM and incubated in the presence or absence of ROT (0.5 μM) for another 24 h (The schematic experimental study plan is depicted in Supplementary Figure S2A). Different passages of SH-SY5Y cells treated with different batches of hADSC-CM or NI-hADSC-CM for three independent experiments. After 48 h, floating cells in medium were combined with adherent cells harvested by scraping, pelleted, and washed twice with PBS. Then, cells were exposed to cell lysate buffer (100 mM Tris–HCl (pH 7.6), 100 mM NaCl, 1% Nonidet P-40, 1% sodium deoxycholate, 0.1% sodium dodecyl sulfate (SDS), and 1% Triton X-100) supplemented with protease and phosphatase inhibitors and incubated for 30 min in ice. After centrifugation at 13,200 rpm for 15 min at 4 °C, the supernatants were collected as the total cell lysates. Protein concentrations were determined using the BCA Protein Assay Kit (Thermo Scientific, Rockford, IL, USA; #23225) following the manufacturer’s instructions. Equal amounts of the proteins (15 μg) were separated on 8–14% SDS-polyacrylamide gels and transferred onto nitrocellulose membranes (Millipore, Bradford, MA, USA; HATF00010). The membranes were washed with PBS containing 0.5% (*v/v*) Tween 20 (PBS-T) followed by blocking with 5% (*v/v*) non-fat dried milk solution prepared in PBS-T and then incubated overnight with primary antibodies at 4 °C. The antibodies (Millipore, Temecula, CA, USA; Abcam, Cambridge, MA, USA; Cell Signaling Technology Inc., Danvers, MA, USA; Santa Cruz Biotechnology, Santa Cruz, CA, USA) used are listed in Supplementary Table S1. After this, the membranes were exposed to secondary antibodies conjugated to horseradish peroxidase for 2–3 h at room temperature (RT) and washed thrice with PBS-T. The signals were detected using an enhanced chemiluminescence (ECL) system (Millipore, Billerica, MA, USA; WBLUR0500) and a LAS 4000 luminescent image analyzer (GE Healthcare, Little Chalfont, UK). The membranes were incubated in Western blot Stripping Buffer (Thermo Scientific, #21059) with constant shaking for 60 min. Using different molecular weight loading controls, β-actin or GAPDH, were used to normalize the expression levels of the interested proteins. For detection with Syn211 antibody, samples loaded on to SDS-PAGE gels were, transferred into nitrocellulose membranes were post-fixed in 0.4% paraformaldehyde (PFA; GeneAll, Seoul, South Korea; SM-P-01-100) in PBS for 30 min followed by three washes with PBS before blocking with milk solution. Densitometric analysis was performed using ImageJ (National Institute of Health, USA) software.

### 4.6. Triton X-100-Soluble and -Insoluble Fractionation and Western Blotting for Evaluating the Expression of α-syn

To assess the α-syn aggregation in SH-SY5Y cells, Western blot analyses of the Triton X-100-soluble and -insoluble (2% SDS soluble) lysate fractions were performed after omitting hADSC-CM groups (The experimental study plan is depicted in Supplementary Figure 3a). After 48 h of experiment, SH-SY5Y cells were lysed in cell lysis buffer containing protease and phosphatase inhibitors with 1% Triton X-100 as mentioned above for 30 min in ice. After centrifugation at 13,200 rpm for 15 min at 4 °C, the supernatants were collected as the Triton X-100-soluble fractions. The cell pellets were washed with PBS, dissolved in the cell lysis buffer containing protease and phosphatase inhibitors with 1% Triton X-100 and 2% SDS, and then used as Triton X-100-insoluble fraction after sonication on ice six times for 10 s each. Protein concentrations were determined by BCA Protein Assay Kit. Equal amounts of the proteins (30 μg) were separated on 8 or 12% SDS-polyacrylamide gels and transferred onto nitrocellulose membranes. Immediately after the transfer, the membranes were pre-fixed with 4% PFA in PBS containing 0.01% glutaraldehyde (Sigma 340855) for 60 min at RT and then washed with PBS. Blocking was performed with 5% skim milk in Tris-buffered saline (TBS) with 0.1% Tween-20 (TBS-T) for 60 min. Membranes were then incubated with anti-p-S129 α-syn (Abcam, ab51253) primary antibody diluted in blocking buffer overnight at 4 °C. The membranes were then washed thrice for 10 min each in TBS and incubated with the secondary antibodies for 3 h diluted in blocking buffer. After washing the membranes thrice for 10 min each in TBS, the signals were visualized using ECL. After visualizing p-S129 α-syn, the membranes were washed with PBS-T and incubated in Western blot Stripping Buffer with constant shaking for 60 min. After being washed with PBS-T thrice for 10 min each, the membranes were pre-fixed with 4% PFA in PBS for 60 min at RT and then rinsed with PBS. After blocking in 5% skim milk in TBS-T for 60 min, the membranes were then incubated with total α-syn (Abcam, ab212184) primary antibody diluted in blocking buffer overnight at 4 °C. Subsequently, the membranes were washed thrice for 10 min each in TBS and incubated with secondary antibodies diluted in blocking buffer. Next, the membranes were washed thrice for 10 min each in TBS, and the signals were visualized using ECL. As β-actin bands are very faint (data not shown) due to membrane fixation, another loading control GAPDH was used to normalize the protein expression levels. Densitometric analysis was performed using ImageJ software.

### 4.7. Statistical Analysis

Data are expressed as the mean ± standard error mean (SEM). The statistical significance of the effects of the treatments was determined using one-way analysis of variance (ANOVA) followed by Tukey’s post hoc multiple comparison test. Differences with *p* values <0.05 were considered statistically significant. GraphPad Prism^®^ 5.0 software (GraphPad Software Inc., San Diego, CA, USA) was used for analyzing data and plotting graphs.

## 5. Conclusions

**Figure 8:**
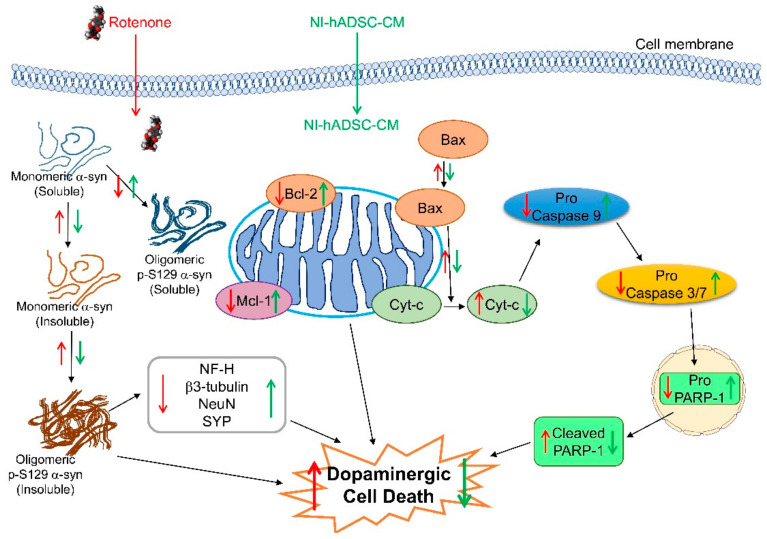
Diagrammatic representation of various activities of NI-hADSC-CM in the ROT-induced PD-like model. The downward arrows (↓) denote inhibitions, and the upward arrows (↑) denote stimulation by ROT (red) and NI-hADSC-CM (green).

In summary, the ROT-induced decrease in cell survival was ameliorated by NI-hADSC-CM treatment. NI-hADSC-CM increased the ROT-induced depletion of tyrosine hydroxylase (TH) protein. The ROT-induced phosphorylation and aggregation of soluble α-syn into insoluble α-syn was prevented by NI-hADSC-CM treatment; NI-hADSC-CM reduced α-syn phosphorylation, blocked the formation of toxic α-syn oligomers, and stabilized the soluble α-syn monomers. NI-hADSC-CM treatment improved the neuronal function by inhibiting the ROT-induced suppression of the expression levels of the NF-H, β3-tubulin, NeuN, and SYP proteins. Mitochondrial dysfunction was detected by evaluating the changes in the Bax/Bcl-2 ratio and decreased Mcl-1 expression levels, which resulted in the activation of caspases-9, -3, and -7, and PARP-1. NI-hADSC-CM treatment regulated the ratio of Bax/Bcl-2, upregulated the expression of the pro-caspases -9, -3, and -7, and inactivated PARP-1. These results indicated that NI-hADSC-CM exerts therapeutic effects against PD through the inhibition of cell death, stabilization of α-syn monomers, promotion of neurogenesis, and suppression of apoptosis (Figure 8). These results hypothesize that the reduction of the levels of insoluble oligomeric p-S129 and total α-syn, along with the preservation of the levels of soluble α-syn, may be sensitive markers for the treatment of PD. Moreover, hADSC-CM treatment decreased the cell numbers and have no effect against ROT toxicity on SH-SY5Y cells evidenced that NI-hADSC-CM have more beneficial effects compared to the effects of hADSC-CM. These therapeutic effects of NI-hADSC-CM may be due to the release of several biological molecules into the conditioned medium during the bFGF- and forskolin-induced neural differentiation. In our future studies, we will attempt to identify the released bioactive factors/molecules in NI-hADSC-CM that are responsible for its neuroregenerative potential. Additionally, it is necessary to study the signaling pathways present downstream of α-syn for developing safer therapeutic strategies.

## Supplementary Materials

The following are available online at https://www.mdpi.com/1422-0067/22/5/2322/s1.
