# Supplementary material for: Neural-Induced Human Adipose Tissue-Derived Stem Cells Conditioned Medium Ameliorates Rotenone-Induced Toxicity in SH-SY5Y Cells
Source: Int J Mol Sci. 2021 Feb 26;22(5):2322. doi: 10.3390/ijms22052322 (PMC7956615; doi:10.3390/ijms22052322)
Supplement: Supplementary file 1 [file ijms-22-02322-s001.pdf]

Supplementary materials to

Neural-Induced Human Adipose Tissue-Derived Stem Cells Conditioned Medium Ameliorates Rotenone-Induced Toxicity in SH-SY5Y Cells

Mahesh Ramalingam, Sujeong Jang, and Han-Seong Jeong

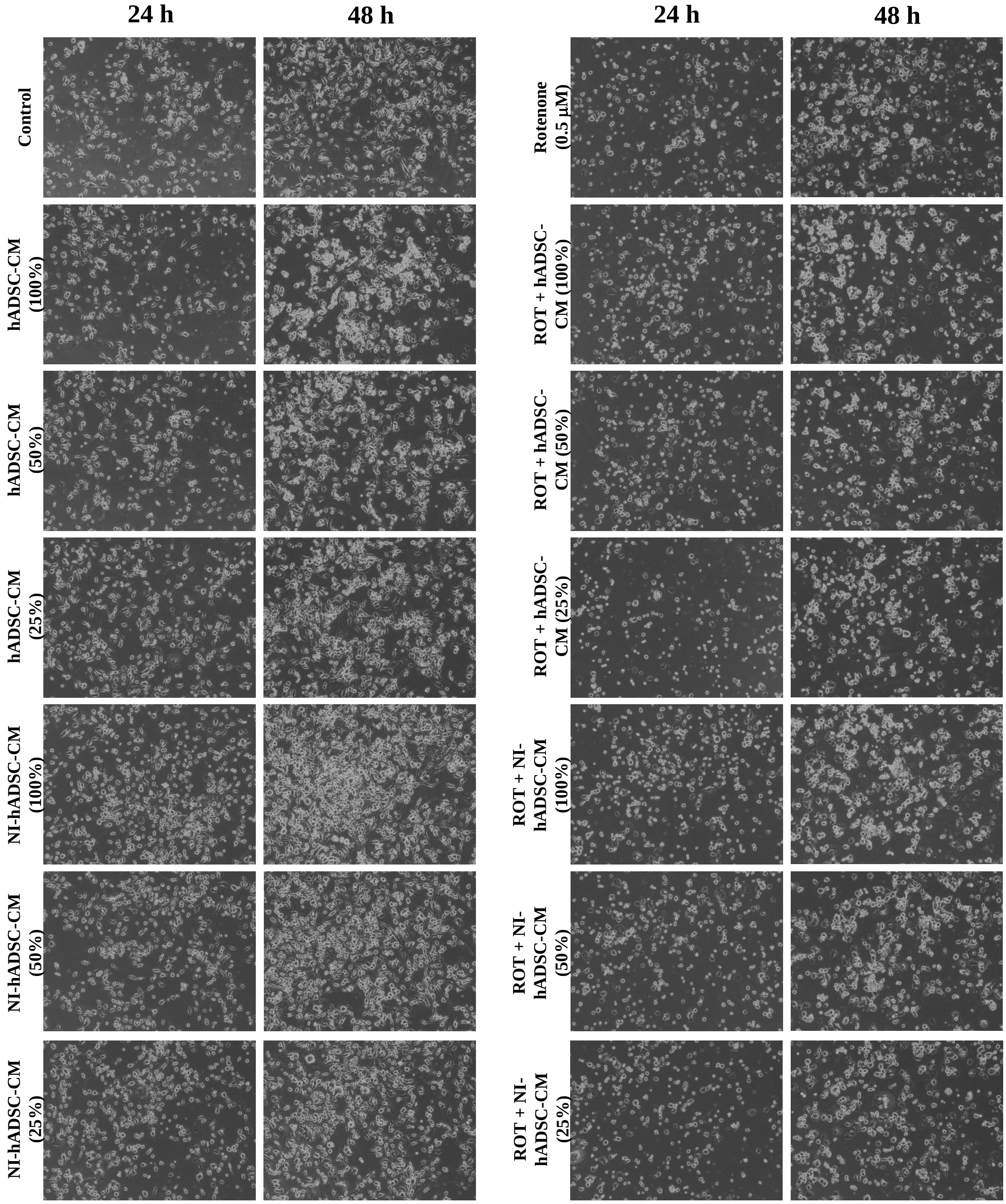

Supplementary Figure 1. SH-SY5Y cells were seeded as 5×10^4 cells/ml of DMEM containing 1% FBS and used for experiments after overnight incubation. Cells incubated with the absence or presence of ROT (0.5 µM) for 48 h were treated with hADSC-CM or NI-hADSC-CM at 100 or 50 or 25% during the last 24 h and assessed for morphological changes. Each picture is a representative of three independent experiments.

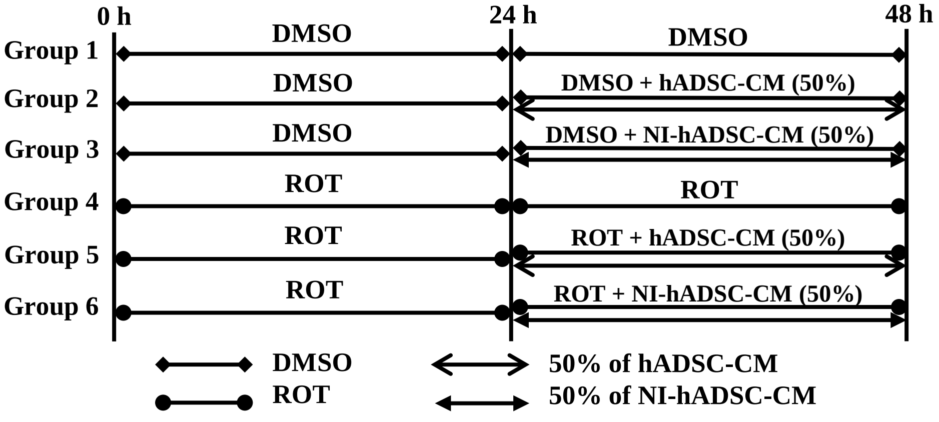

(a)

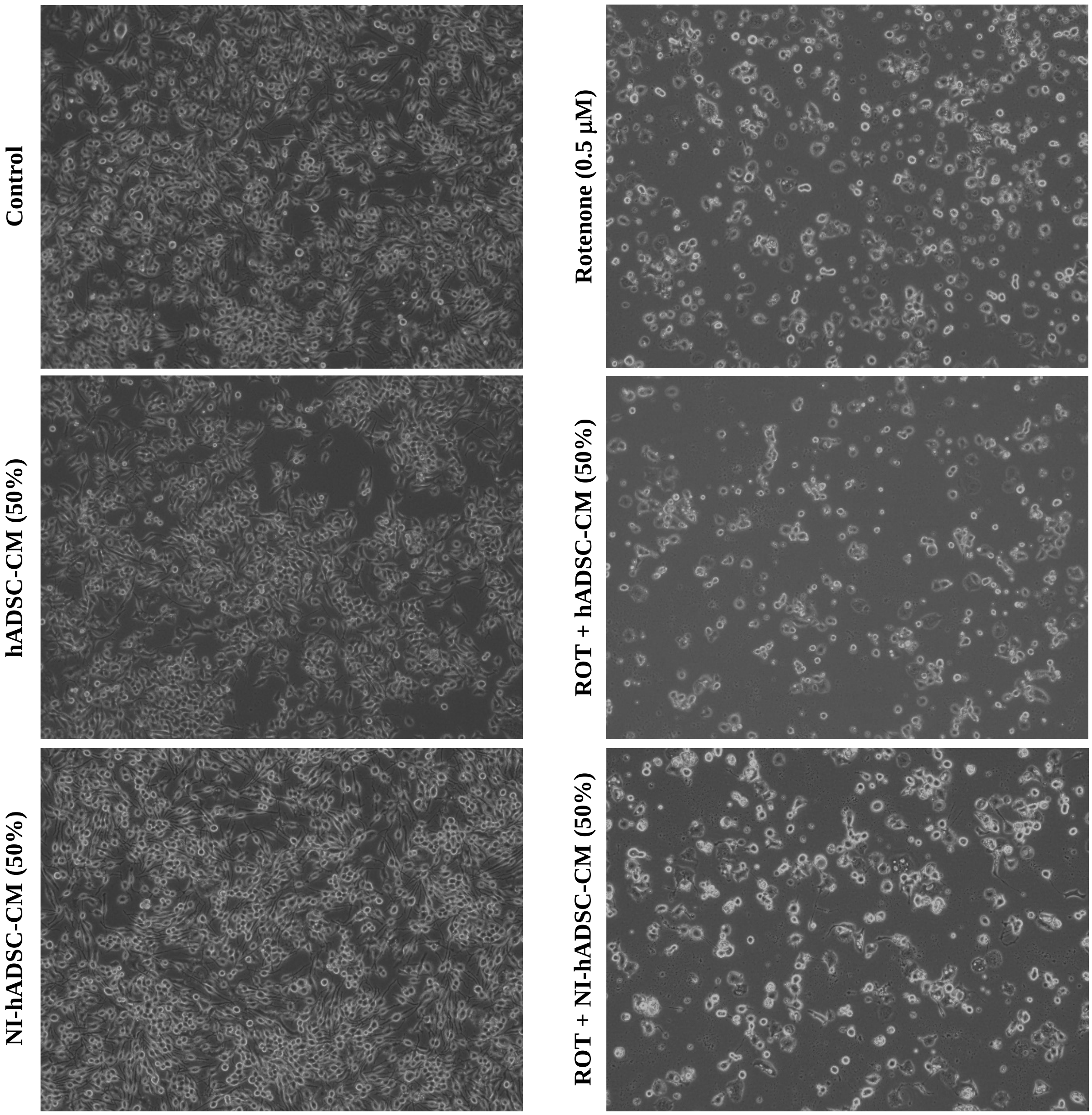

(b)

Supplementary Figure 2. (a) The experimental study plan. (b) SH-SY5Y cells were seeded as 5×10^4 cells/ml of DMEM containing 1% FBS and used for experiments after overnight incubation. Cells incubated with the absence or presence of ROT (0.5 μM) for 48 h were treated with hADSC-CM (50%) or NI-hADSC-CM (50%) during the last 24 h and assessed for morphological changes. Images are representative of three independent experiments.

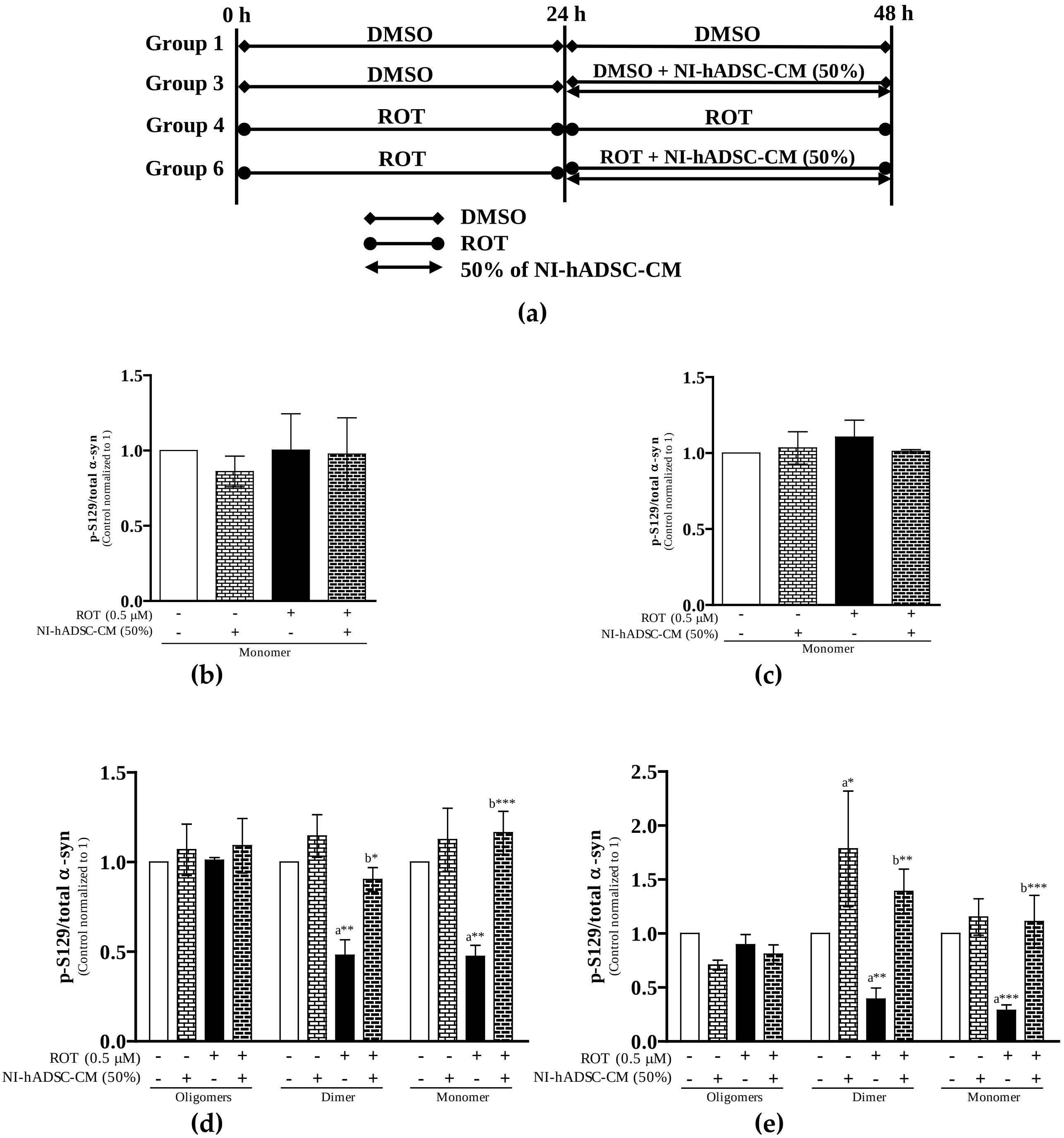

Supplementary Figure 3. The experimental study plan for Triton X-100-soluble and –insoluble fractionation and Western blotting (a). SH-SY5Y cells were seeded as 5×10^4 cells/ml of DMEM containing 1% FBS and used for experiments after overnight incubation. Cells with absence or presence of ROT (0.5 μM) for 48 h were treated with hADSC-CM (50%) or NI-hADSC-CM (50%) during the last 24 h and analyzed by Western blotting. Bar graphs represents fold changes in monomeric p-S129/total α-syn ratios from SDS-PAGE gels of 12% (b) or 8% (c) in Triton X-100-soluble fraction. The oligomeric, dimeric and monomeric S129/total α-syn ratios from SDS-PAGE gels of 12% (d) or 8% (e) in Triton X-100-insoluble fraction. Data are mean ± SEM of three independent experiments and analyzed by one-way of variance (ANOVA) followed by Tukey's *post hoc* test. Statistical significance: ^{a}-compared with control; ^{b}-compared with ROT; *p<0.05 and ***p<0.001.

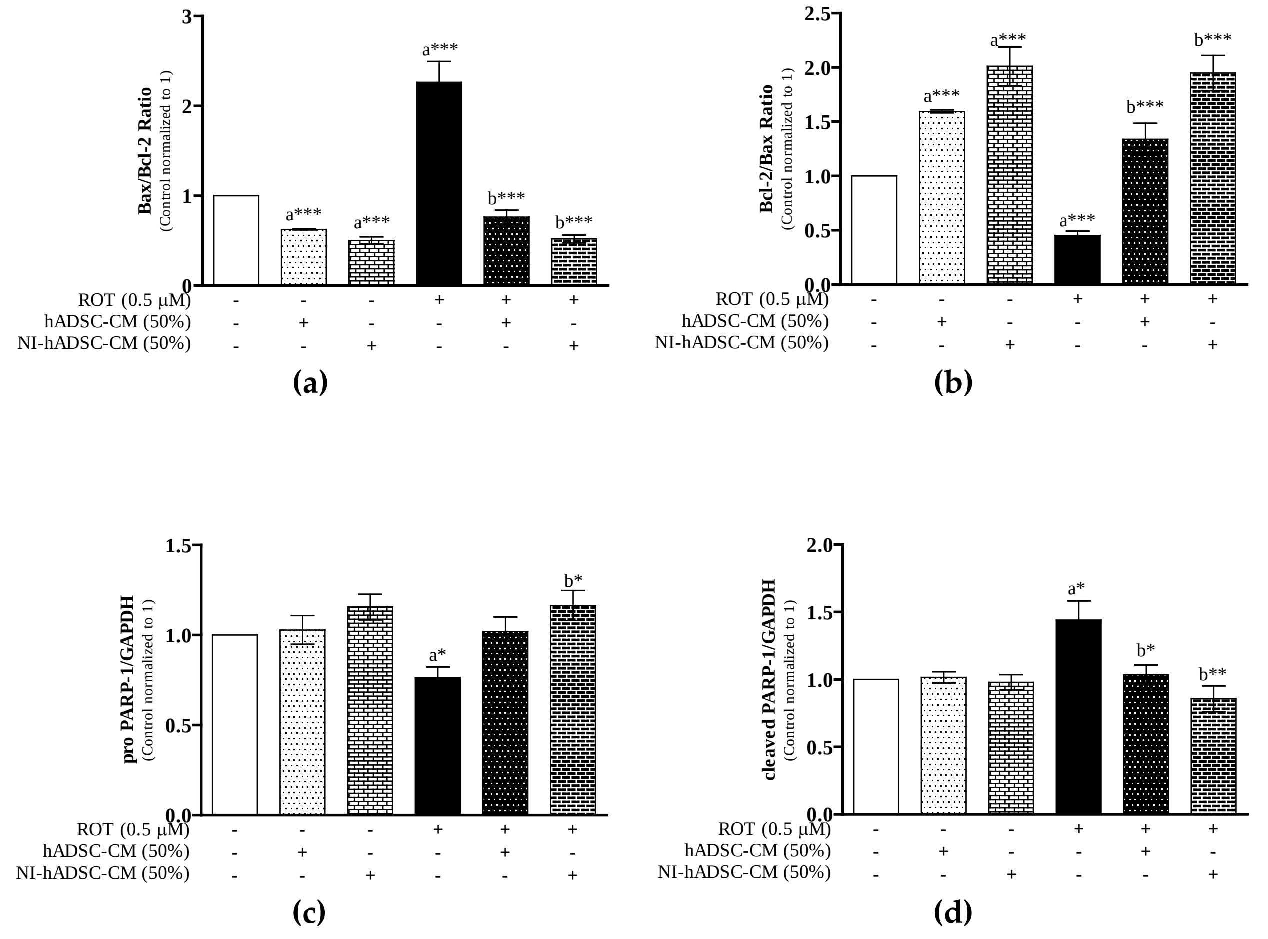

Supplementary Figure 4. SH-SY5Y cells were seeded as 5×10^4 cells/ml of DMEM containing 1% FBS and used for experiments after overnight incubation. Cells with absence or presence of ROT (0.5 μM) for 48 h were treated with hADSC-CM (50%) or NI-hADSC-CM (50%) during the last 24 h and analyzed by Western blotting. The bar graphs represents for Bax/Bcl-1 ratio (a), Bcl-2/Bax ratio (b), pro-PARP-1/GAPDH (c) and cleaved PARP-1/GAPDH ratio (d). Images are representative of three independent experiments. Data are mean ± SEM of three independent experiments and analyzed by one-way of variance (ANOVA) followed by Tukey's *post hoc* test. Statistical significance: [a]-compared with control; [b]-compared with ROT; *p<0.05, **p<0.01 and ***p<0.001.

Supplementary Table 1. Western blotting conditions and antibodies used in this study.

Western blotting conditions:

SDS-PAGE Gel Percentages

8%	= TH, p-S129 α-syn, total α-syn, NF-H, PARP
12%	= p-S129 α-syn, total α-syn, β3-tubulin, NeuN, SYP
13 or 14%	= Syn211, Bax, Bcl-2, Mcl-1, Cyt-c, Cas-9, -3, -7.

SDS-PAGE Gel Running:

80~100 V for 100~120 min

SDS-PAGE Gel Transfer Times to Nitrocellulose Membrane:

8%	= 250 mA for 90 min
12%	= 200 mA for 65 min
13 or 14%	= 200 mA for 60 min

Antibody Name	Host, MW Details	Company	Cat. No.	Dilution
**Primary Antibodies:**				
Tyrosine hydroxylase	Rabbit pAb, 62 kDa	Millipore	AB152	1:1,000
α-synuclein clone Syn211	Mouse mAb, 14 kDa	Millipore	36-008-25UL	1:1,000
p-S129 α-synuclein	Rabbit mAb, 18 kDa	Abcam	ab51253	1:1,000
total α-synuclein	Rabbit mAb, 18 kDa	Abcam	ab212184	1:1,000
Neurofilament-H	Mouse mAb, 180~200 kDa	Cell Signaling	#2836	1:1,000
β3-tubulin	Rabbit mAb, 55 kDa	Cell Signaling	#5568	1:1,000
Neuronal Nuclei	Mouse mAb, 46~48 kDa	Millipore	MAB377	1:1,000
Synaptophysin	Mouse mAb, 38~48 kDa	Santa Cruz	sc-17750	1:2,000
Bax	Rabbit pAb, 23 kDa	Santa Cruz	sc-493	1:500
Bcl-2	Rabbit pAb, 26 kDa	Santa Cruz	sc-492	1:500
Mcl-1	Rabbit mAb, 40 kDa	Cell Signaling	#94296	1:1,000
Cytochrome c (Cyt-c)	Rabbit mAb, 14 kDa	Cell Signaling	#11940	1:1,000
Caspase-9	Mouse mAb, pro=47, cleaved=37,35 kDa	Cell Signaling	#9508	1:1,000
Caspase-3	Rabbit mAb, pro=35, cleaved=17,19 kDa	Cell Signaling	#9665	1:1,000
Caspase-7	Rabbit mAb, Pro=35, cleaved=20 kDa	Cell Signaling	#12827	1:1,000
PARP	Rabbit pAb, Pro=116, cleaved=89 kDa	Cell Signaling	#9542	1:1,000
GAPDH	Rabbit pAb, 37 kDa	Santa Cruz	sc-25778	1:2,000
β-actin	Mouse mAb, 43 kDa	Santa Cruz	sc-47778	1:2,000
**Secondary Antibodies:**				
Anti-rabbit IgG, HRP-linked antibody		Cell Signaling	#7074	1:1,000 ~1:2,000
Anti-mouse IgG, HRP-linked antibody		Cell Signaling	#7076	1:1,000 ~1:2,000

pAb, polyclonal antibody; mAb, monoclonal antibody; kDa, kiloDalton.

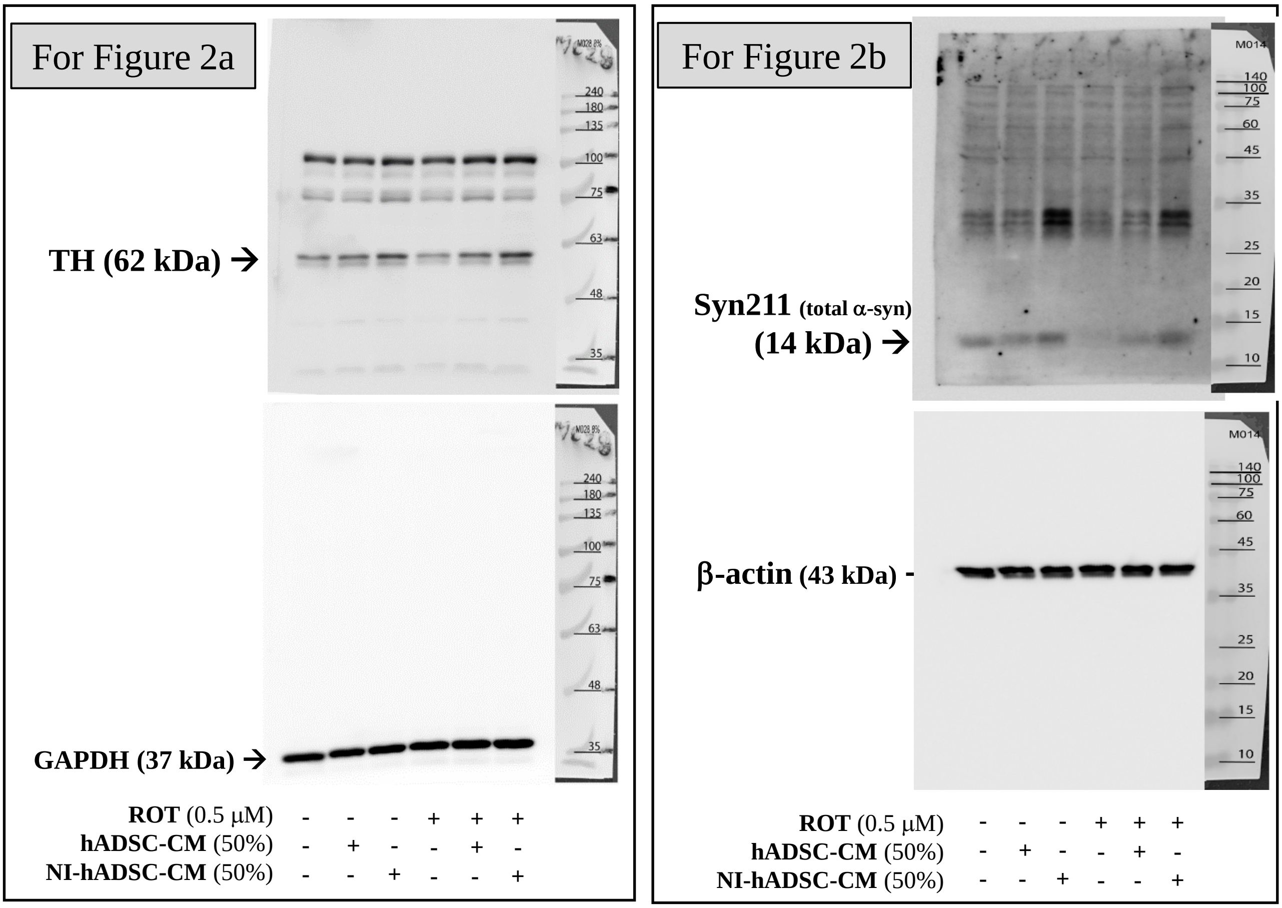

Supplementary Figure 5. Unedited images and their molecular weight markers for respective Western blots used in **Figure 2** of this manuscript.

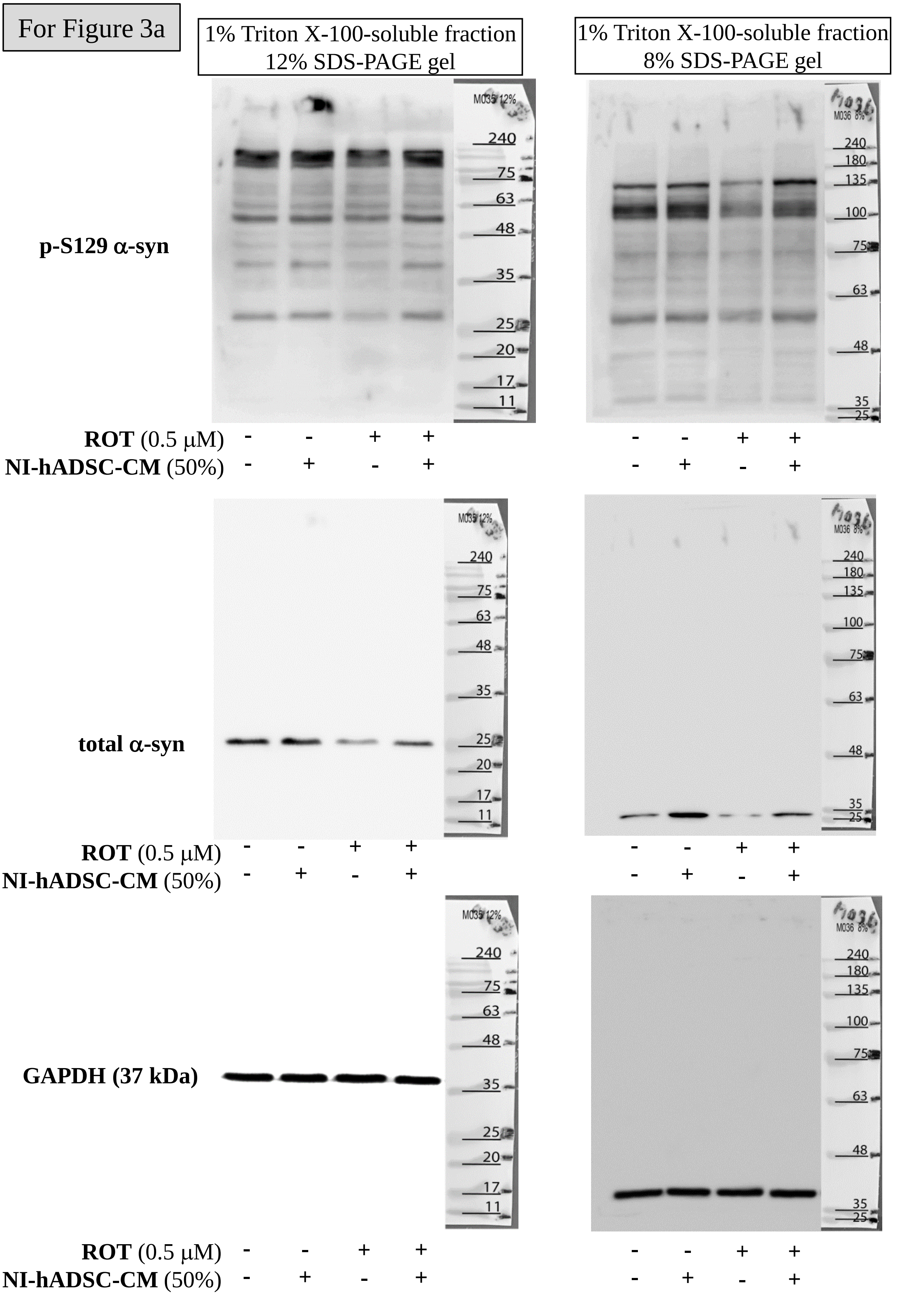

Supplementary Figure 6. Unedited images and their molecular weight markers for respective Western blots used in **Figure 3a** of this manuscript.

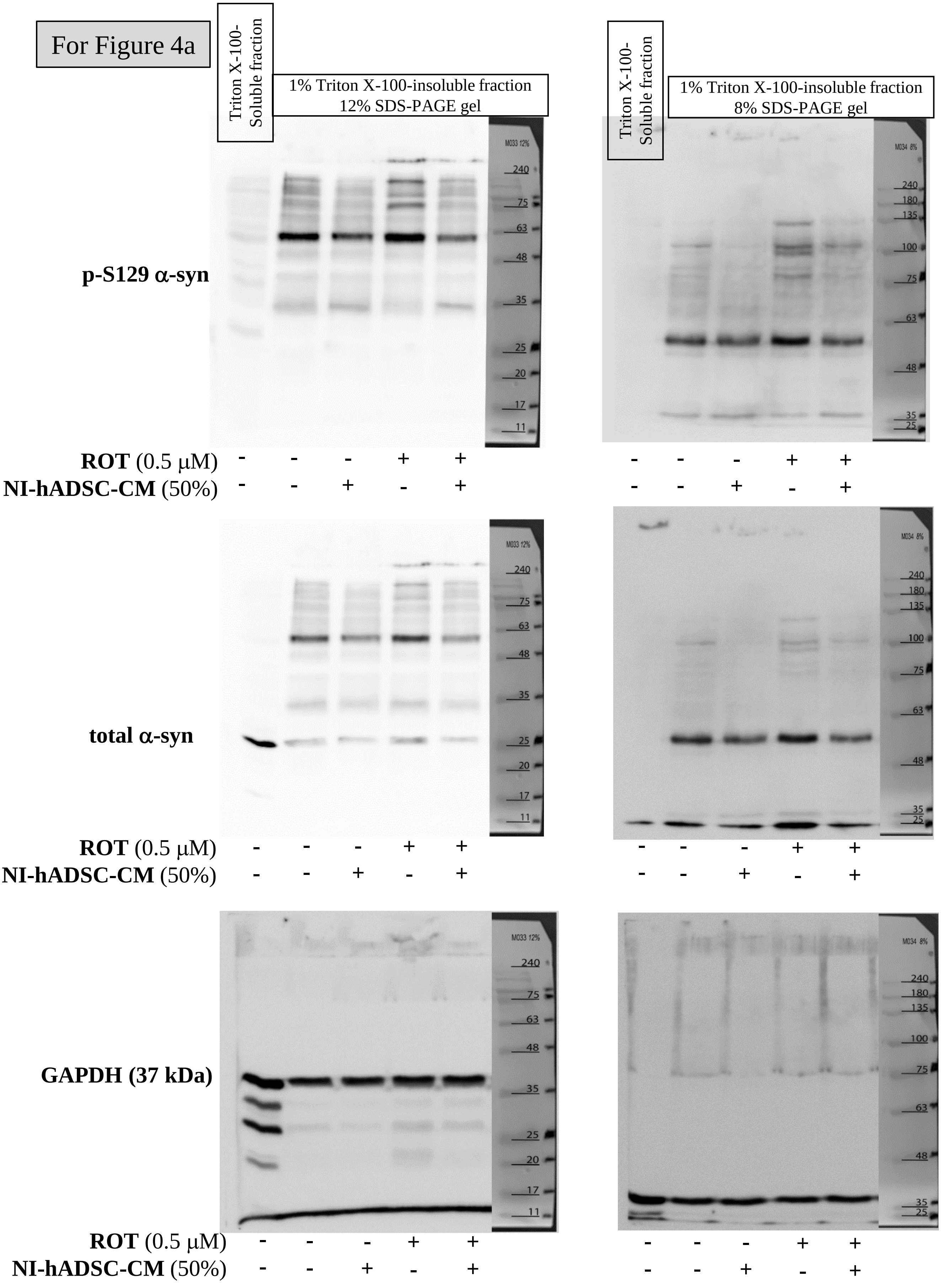

Supplementary Figure 7. Unedited images and their molecular weight markers for respective Western blots used in **Figure 4a** of this manuscript.

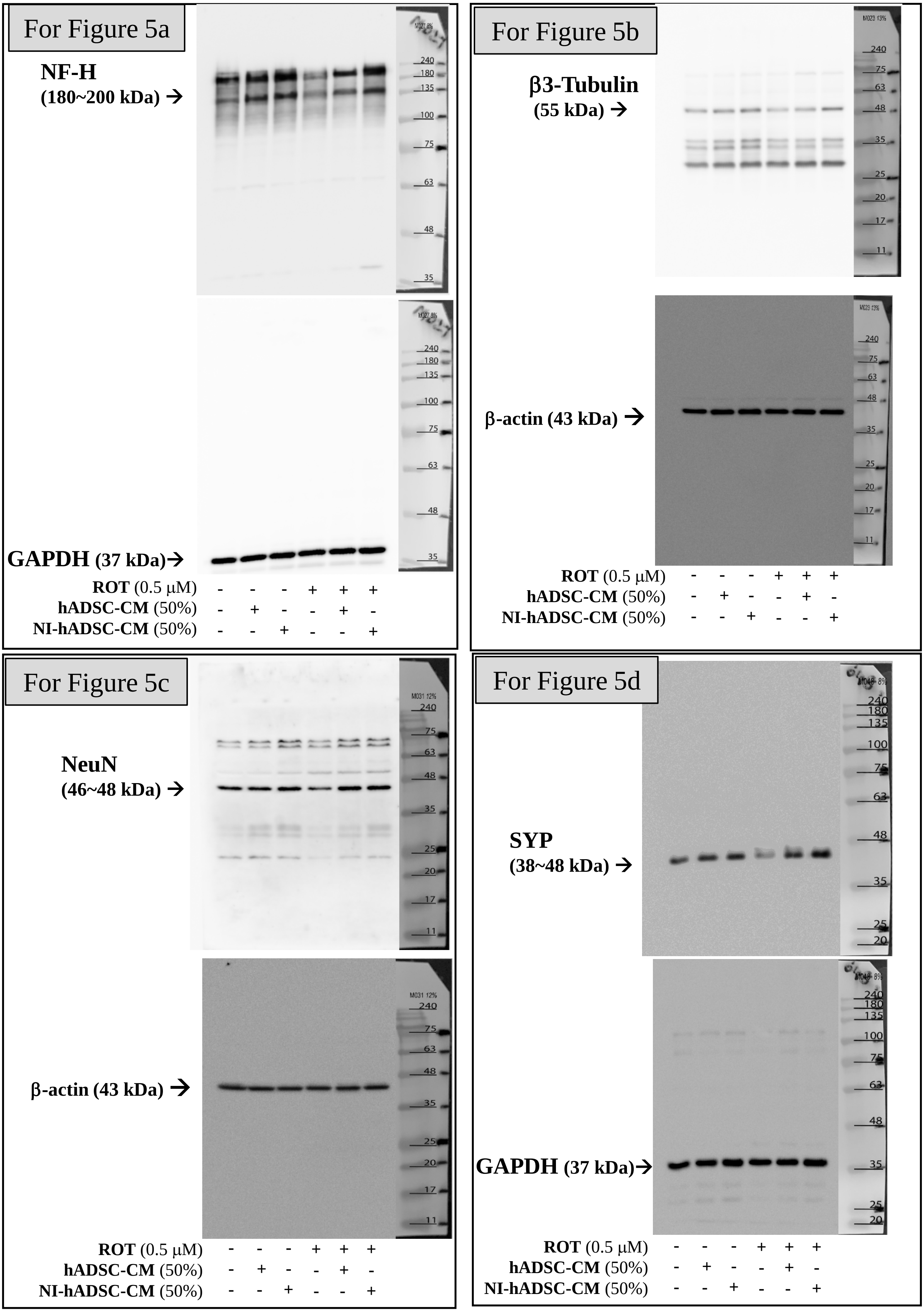

Supplementary Figure 8. Unedited images and their molecular weight markers for respective Western blots used in **Figure 5** of this manuscript.

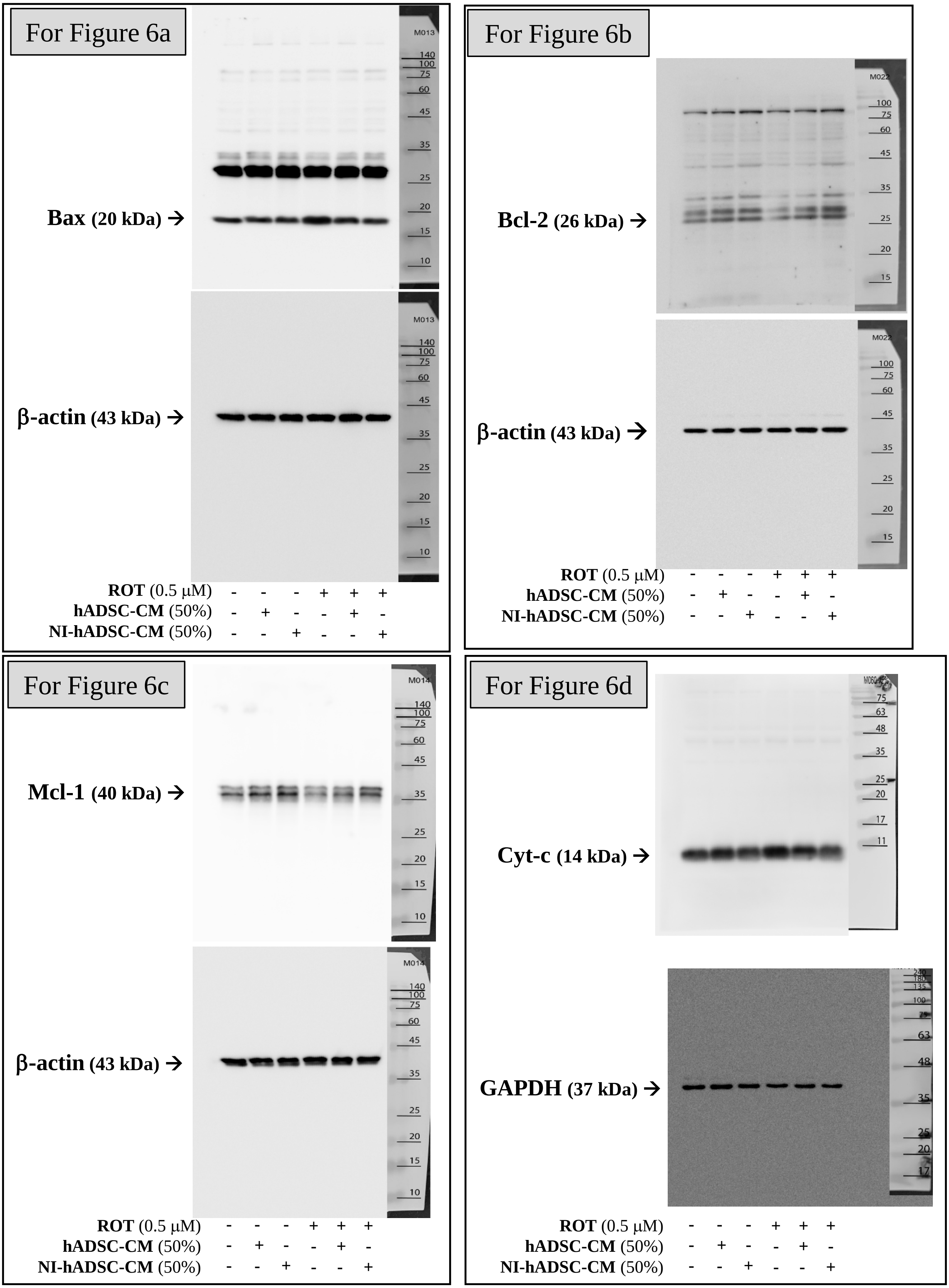

Supplementary Figure 9. Unedited images and their molecular weight markers for respective Western blots used in **Figure 6** of this manuscript.

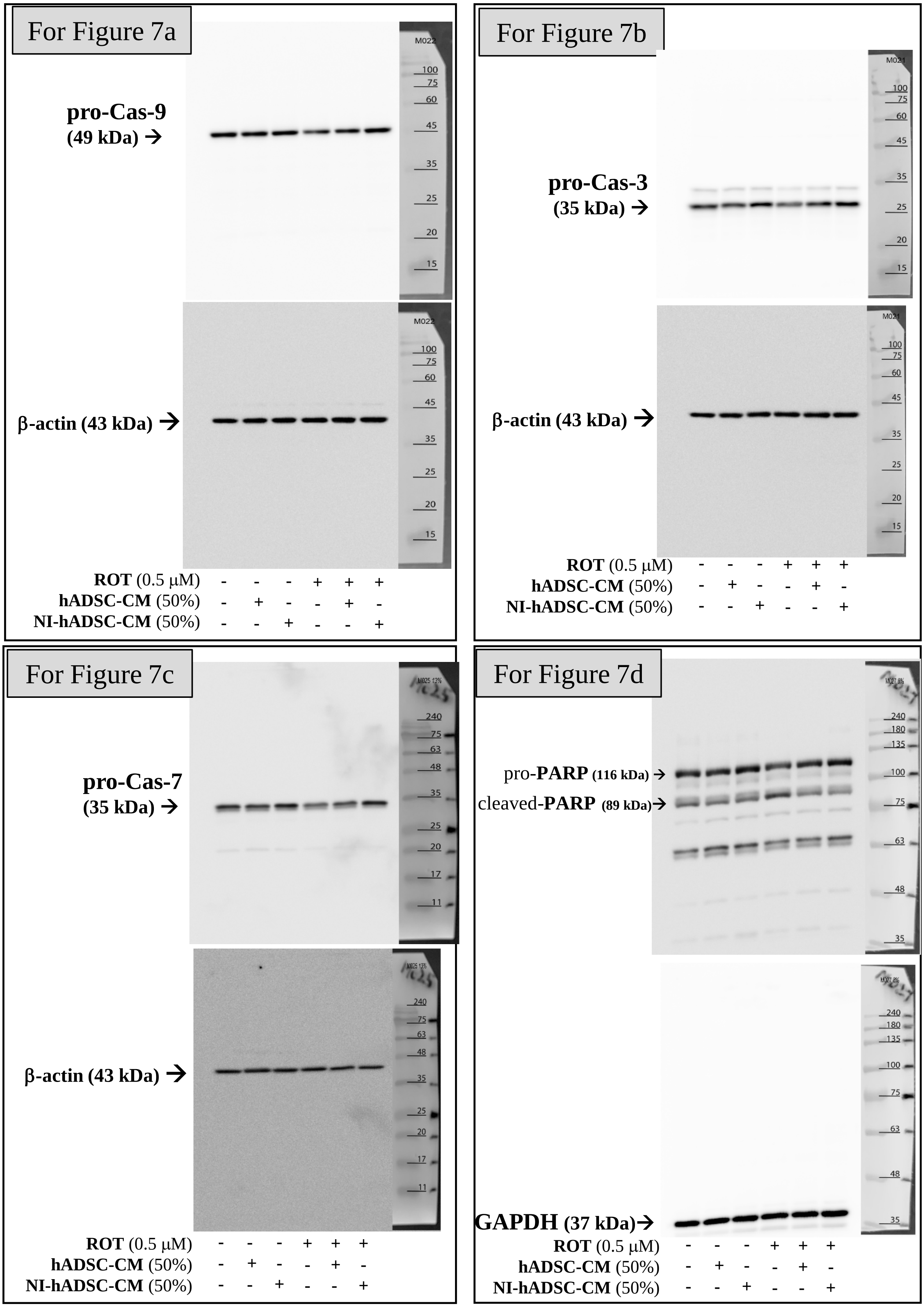

Supplementary Figure 10. Unedited images and their molecular weight markers for respective Western blots used in **Figure 7** of this manuscript.
